# Intramolecular Charge Transfer and Spin–Orbit Coupled Intersystem Crossing in Hypervalent Phosphorus(V) and Antimony(V) Porphyrin Black Dyes

**DOI:** 10.1021/jacs.4c06674

**Published:** 2024-09-09

**Authors:** Jam Riyan Hamza, Jatan K. Sharma, Paul A. Karr, Art van der Est, Francis D’Souza, Prashanth K. Poddutoori

**Affiliations:** † Department of Chemistry & Biochemistry, University of Minnesota Duluth, Duluth, Minnesota 55812, United States; ‡ Department of Chemistry, University of North Texas, Denton, Texas 76203-5017, United States; § Department of Chemistry, Brock University, St. Catharines, Ontario L2S 3A1, Canada; ∥ Department of Physical Sciences and Mathematics, Wayne State College, 1111 Main Street, Wayne, Nebraska 68787, United States

## Abstract

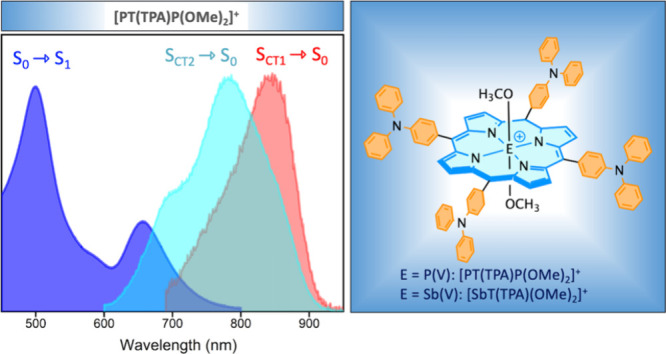

Porphyrin dyes with strong push–pull type intramolecular charge transfer (ICT) character and broad absorption across the visible spectrum are reported. This combination of properties has been achieved by functionalizing the periphery of hypervalent and highly electron-deficient phosphorus(V) and antimony(V) centered porphyrins with electron-rich triphenylamine (TPA) groups. As a result of the large difference in electronegativity between the porphyrin ring and the peripheral groups, their absorption profiles show several strong charge transfer transitions, which in addition to the porphyrin-centered π → π* transitions, make them panchromatic black dyes with high absorption coefficients between 200 and 800 nm. Time-resolved optical and electron paramagnetic resonance (EPR) studies show that the lowest triplet state also has ICT character and is populated by spin–orbit coupled intersystem crossing.

The properties of the chromophore govern the function of any photoactive molecular complex.^1−3^ Thus, the successful design of any such system requires synthetic control of these properties. While controlling a given property is relatively straightforward, optimizing a desired set of properties is challenging. Among the chromophores, porphyrin molecules have received much attention because of their tunable structure, which enables the optimization of the desired properties for various applications.^4−11^ A long-standing goal in solar energy conversion has been designing push–pull type photoinduced intramolecular charge transfer (ICT) complexes that absorb a broad range of wavelengths and have long-lived CT excited states.^12−19^ Our efforts in this area have focused on the main group element porphyrins.

The group 15 elements P(V) or Sb(V) are exciting because they induce very high reduction potentials in the porphyrin and can accommodate axial ligands.^20−24^ We and others have shown that these porphyrins perform well in donor–acceptor systems that exhibit light-induced electron transfer.^25−36^ However, the yield of charge separation in these complexes is limited by the relatively narrow absorption bands of the porphyrin and competing processes, such as intersystem crossing (ISC), luminescence, and charge recombination. These limitations prompted us to investigate whether attaching strong electron donors to the periphery of the P(V) porphyrins and Sb(V) porphyrins could induce CT character in their lowest excited states. We reasoned that this would result in broadening and additional transitions in the absorption spectrum and might lead to the population of the CT state, which would help to improve the efficiency of subsequent electron transfer. The inherent directionality of electron migration and charge transfer (CT) can be utilized for various applications.^14,15,37−42^ For instance, in artificial photosynthetic systems such as dye-sensitized solar cells and photocatalytic cells, which are becoming increasingly important.

With this objective, we synthesized two novel porphyrins with electron-rich triphenylamine (TPA) substituents attached to the four *meso*-positions (Scheme S1). These compounds [PT(TPA)P(OMe)_2_]Cl (**1**) and [SbT(TPA)P(OMe)_2_]PF_6_ (**2**) and their reference systems [PTPP(OMe)_2_]PF_6_ (**3**) and [SbTPP(OMe)_2_]PF_6_ (**4**) are depicted in Figure 1. The synthesis and structural characterizations of **1** and **2** are described in the SI, and the NMR and mass spectrometry data confirm their structures (see Figures S1–S4). As shown in Figure 1 and Table S1, the absorption spectra and optical data of **1** and **2** in CH_3_CN differ dramatically from those of the corresponding reference compounds **3** and **4**. The B-bands near 400 nm are blue-shifted by 9–18 nm, but the most striking difference is seen in the visible region. While the reference porphyrins show two Q-bands around 550 nm that are components of a vibronic series, compounds **1** and **2** have two stronger and very broad bands that span the region from 450 to 800 nm, making them panchromatic black dyes (Figure 1 inset). Similar behavior has been observed in two related systems reported recently in the literature.^43,44^ DFT calculations of the cationic parts of **1** and **2** show that the LUMO and LUMO+1 are localized primarily on the porphyrin ring while the HOMO and HOMO–1 are delocalized over the four TPA substituents; see Figure 1C bottom left and Figure S5. Electrochemical studies also confirm this (Figure S6 and Table S2). Thus, transitions from the HOMO and HOMO–1 to the LUMO and LUMO+1 lead to ICT from the TPA substituents to the porphyrin ring, as illustrated by the natural transition orbitals in Figure S7. Time-dependent DFT calculations also reveal that, in addition to the usual Q- and B-band states, eight low-lying CT excited states with differing oscillator strengths contribute to the absorption spectra (Figure S8 and Tables S3 and S4). The ICT nature of the lowest energy states is borne out by the electrostatic potential (ESP) and difference density (DD) maps (Figure 1C and Figures S9–S12). The ESP maps show that the porphyrin ring is strongly electropositive in the ground state (blue region) and the TPA groups are electronegative (red regions). The DD maps show that excitation to the eight lowest excited states leads to electron density loss (red) on the TPA groups and gain (green) on the porphyrin ring. None of the ICT absorption bands show a strong dependence on the solvent polarity (Figure S13), probably because the charge distribution in the excited states is symmetric, which means that the excitation does not cause a large change in the dipole moment.

**Figure 1:**
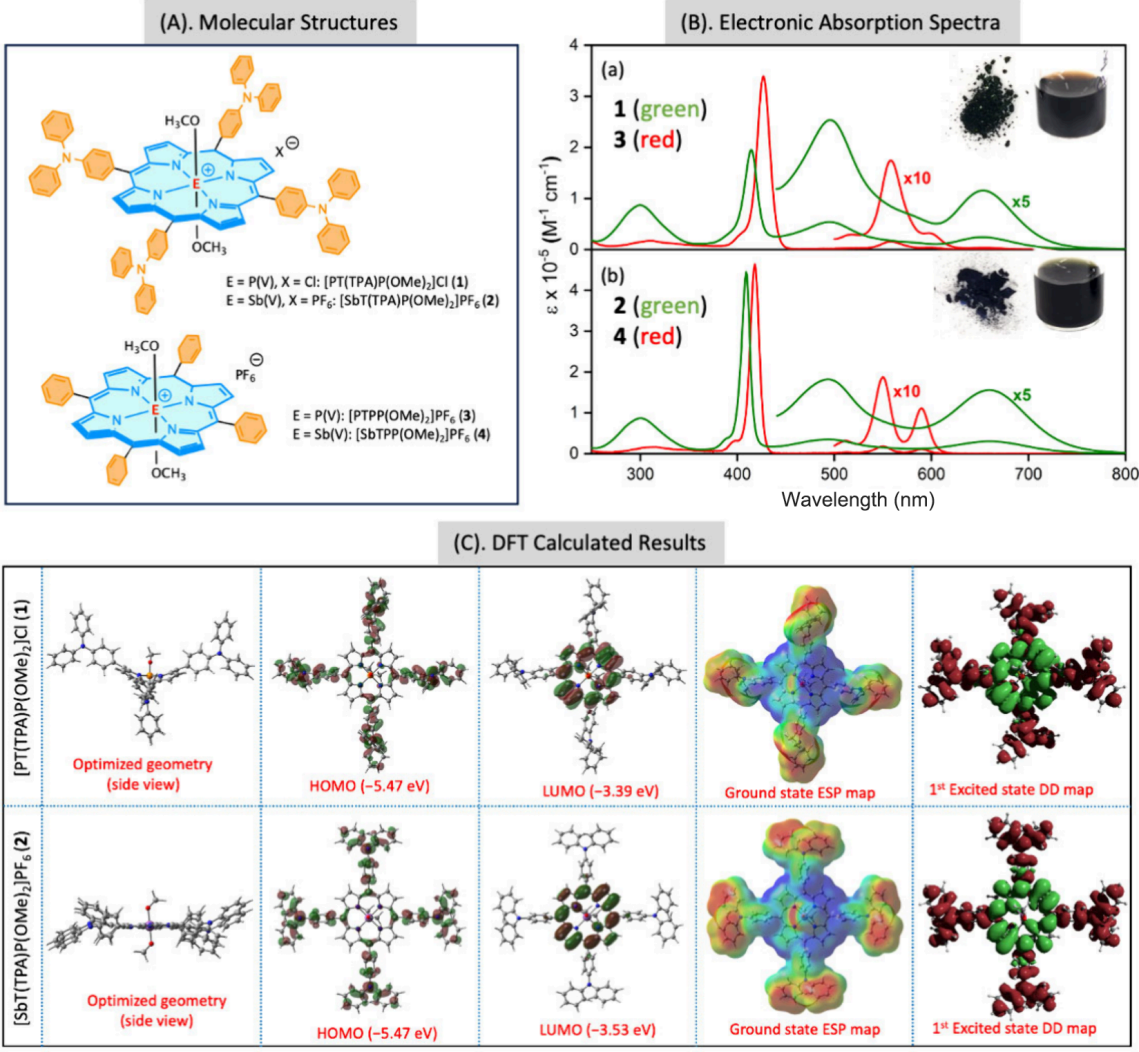
(A) Molecular structures. (B) Absorption spectra of (a) **1** (green) and **3** (red) and (b) **2** (green) and **4** (red) in CH_3_CN at room temperature. (C) DFT calculated results.

The luminescence of **1** and **2** in solvents of different polarity at room temperature is shown in Figure S14. The presence of ICT states leads to a very strong quenching of the fluorescence. Excitation of **1** at 500 nm (Q-band/CT band) gives a broad fluorescence spectrum between 600 and 950 nm. In contrast, excitation at 680 nm (lowest energy CT band) results in an emission that spans the region from 700 to 950 nm. In both cases, the fluorescence intensity is strongly solvent-dependent; in polar CH_3_OH and CH_3_CN, the compounds are nonfluorescent, whereas in nonpolar benzene and toluene, they are weakly fluorescent. In contrast, in moderately polar CH_2_Cl_2_, the emission at 680 and 770 nm vanishes and a band at 850 nm appears with a shoulder at 750 nm. These observations are difficult to explain entirely because of the large number of low-lying states. Still, they can be partially rationalized by proposing that (i) the observed spectra include emission from localized porphyrin singlet states, the eight low-lying CT singlet states, and possibly from the eight CT triplet states and (ii) the dependence of the fluorescence intensity on solvent polarity and the shift of the emission to longer wavelength (750/850 nm) in CH_2_Cl_2_ is the result of changes in the energies of the CT states or a change in the decay pathway through the manifold of states. Similar spectral trends were observed in **2** (Figure S14 on the right). However, the emission intensities are much weaker than for **1**, probably because of faster decay of the CT states, which are expected to have lower energy in **2** based on the redox data.

To test whether the recovery of the 750/850 nm emission band in CH_2_Cl_2_ is due to the solvent-heavy atom effect, its intensity was monitored while titrating with CH_2_Br_2_ in CH_2_Cl_2_ (Figures 2a and S15a). The intensity of the band increases with an increasing CH_2_Br_2_ concentration. More complicated behavior is seen with CH_2_Br_2_ in toluene (Figures 2b and S15b). In this case, an additional band at 700 nm is present, which decreases in intensity as CH_2_Br_2_ is added, while the bands at 750 and 850 nm increase. This rise persists until a 6:4 CH_2_Br_2_:toluene ratio is reached, after which the intensity decreases. We postulate that the initial increase is due to an increase in the ISC rate because of the solvent-heavy atom effect, while the decrease as CH_2_Br_2_ is higher is due to an increase in the rate of decay from the singlet CT state to the ground state as the solvent polarity increases. Similar results were found with compound **2**; see Figure S16. Although these titrations suggest that the 850 nm band has some triplet character, this was not fully supported by variable temperature emission, triplet quenching, and TCSPC lifetime measurements; see SI and Figure S17 for details.

**Figure 2:**
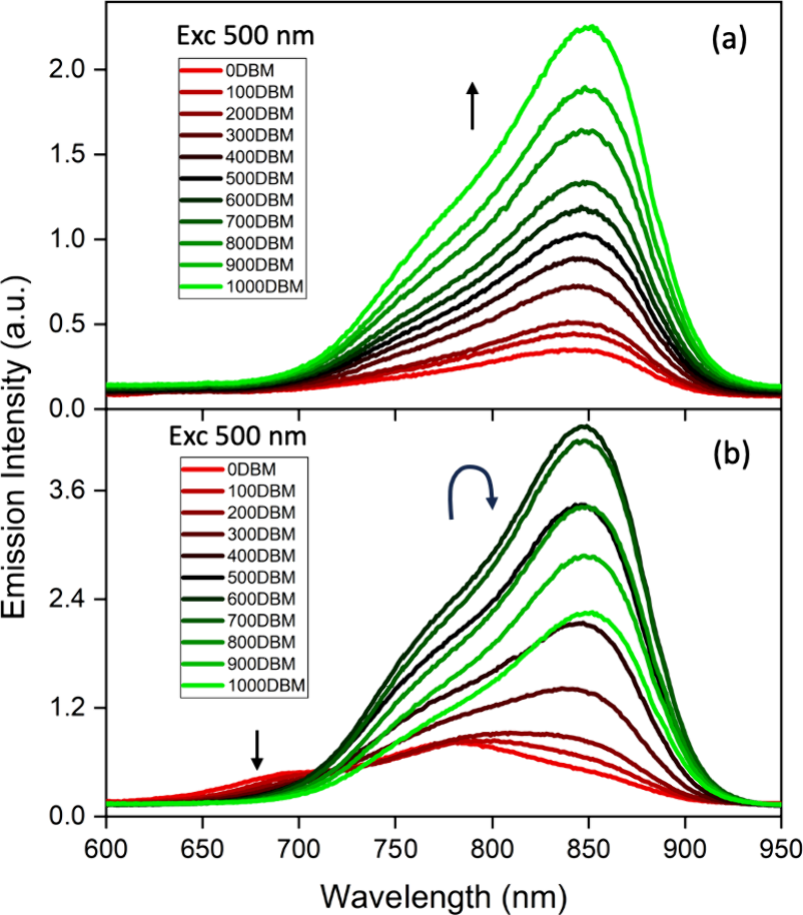
Emission spectra of **1** with increasing volumes (in increments of 100 μL) of CH_2_Br_2_ in (a) CH_2_Cl_2_ and (b) toluene.

Using the absorption and emission maxima, approximate energy level diagrams for **1** and **2** can be constructed, as shown in Figures 3 and S18, along with the normalized spectral overlap of the absorption and emission bands. Based on the energetics, it is apparent that, following excitation, decay to the singlet CT state is expected with subsequent relaxation to either the ground or triplet CT state, depending on the solvent conditions. It is important to note that only the states involved in the absorption and emission are shown in Figure 3. The corresponding diagram from TDDFT calculations (Figure S8) predicts that the singlet and triplet CT states are very close in energy; most likely, some of these states are in equilibrium. Therefore, back-and-forth transitions are possible and are expected to be sensitive to the solvent polarity.

**Figure 3:**
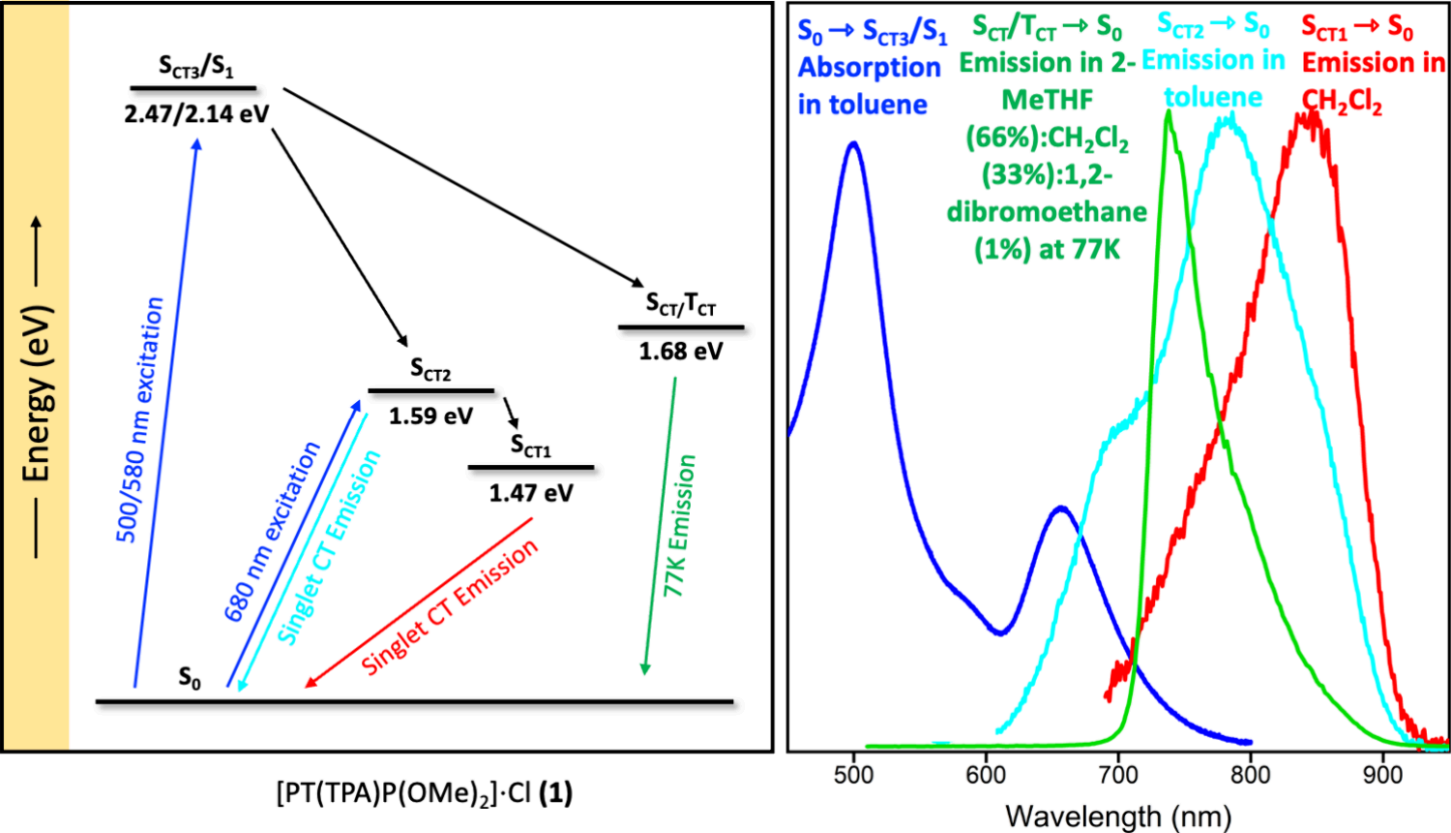
Left: Calculated energy level diagram of **1**. Right: Normalized spectral overlap of **1**.

The femtosecond transient absorption (fs-TA) spectra of **1** in CH_3_CN (Figure 4a) reveal excited state absorption peaks at 452, 602, and 719 nm within the first few ps. Negative peaks at 496 and 640 nm, corresponding to ground-state bleaching, are also observed. The spectra were analyzed using global target analysis, whose decay-associated spectra (DAS) are shown in the middle panel (Figure 4b), while the population time profiles are shown in the right-hand panel (Figure 4c). For **1**, a three-component fit provided satisfactory results. The first two components at τ_1_ = 3.6 ps and τ_2_ = 4.1 ps are complementary to one another and are assigned to the S_1_ (hot) and S_1_ (relaxed) states. The third component at τ_3_ = 228.3 ps has spectral features expected for the CT state (see Figure S19 for the spectra of the oxidized and reduced forms of **1** and **2**). The spectral features observed for **2**, shown in Figure 4d, closely resemble those of **1**. The DAS generated from target analysis required four-component spectra for satisfactory data fitting (Figure 4e). The components at τ_1_ = 0.5 and τ_2_ = 1.0 ps (complementary spectra) could be attributed to the S_2_ and S_1_(hot) states, while the component at τ_3_ = 5.7 ps could be attributed to the relaxed S_1_ state. The fourth component at τ_4_ = 136.7 ps had features expected for the CT state. The CT state lifetime of **1** is slightly longer than that of **2**. Changing the solvent from polar CH_3_CN to nonpolar toluene led to appreciable changes in photodynamics, as shown in Figure S20; see the SI for details.

**Figure 4:**
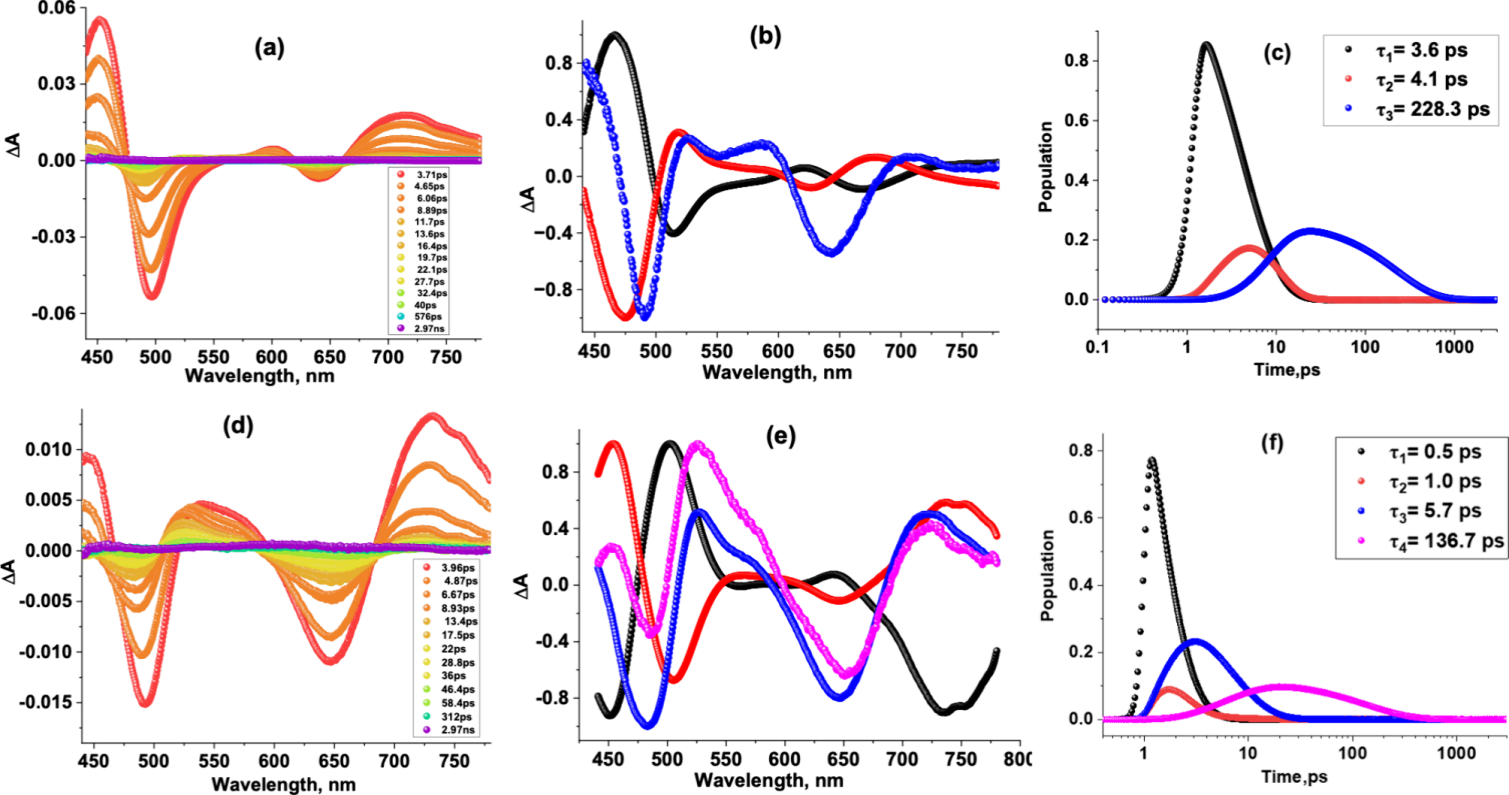
fs-TA spectra of (a) **1** and (d) **2** in CH_3_CN. Their corresponding decay-associated spectra (DAS) (b, e) and the population curves (c, f).

The fs-TA data revealed the formation of CT states of singlet character (^1^CT*) from the S_1_ state. These states are energetically close to the ^3^CT* state, as predicted by TD-DFT calculations (see Figure S8), so spin–orbit coupling mediated ISC between them is possible. Alternatively, the S_1_ state could undergo ISC to populate the energetically low-lying T_1_ state, which is a common photochemical path for organic fluorophores. Since transient absorption spectral differentiation between ^1^CT* and ^3^CT* and between ^3^CT* and T_1_ states is difficult, time-resolved electron paramagnetic resonance (TR-EPR) spectral studies were performed to resolve this issue and seek evidence for forming ^3^CT*.

The CT character of the lowest excited triplet states of **1** and **2** is evident in their TR-EPR spectra (Figure 5). Compared to those of reference compounds **3** and **4**, the widths of the spectra and the value of the zero-field splitting parameter D (Table S6) are reduced by a factor of about 30%. The reduction in D indicates a larger average distance between the unpaired electrons, as is expected when the triplet state has a CT character. The phosphorus(V) porphyrins (**1** and **3)** are both narrower than those of the corresponding antimony(V) porphyrins (**2** and **4**) because the porphyrin ring is significantly nonplanar when substituted with phosphorus (see Figure 1). This is a result of the small size of the P(V) center, and it leads to a spin density distribution that is more spherical and, hence, to a smaller D value than in porphyrins substituted with larger elements, such as Sb. While the spectral widths become smaller, the polarization patterns are not influenced strongly by the change of the substituents from phenyl to TPA, which shows that the ISC continues to be driven by spin–orbit coupling (see the SI).

**Figure 5:**
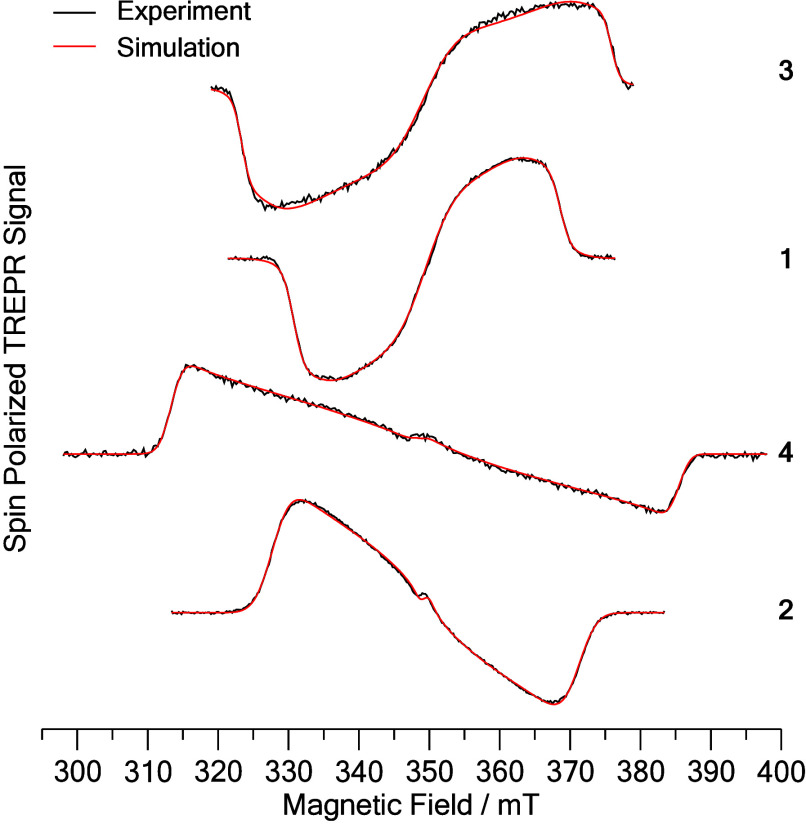
Experimental and simulated spin-polarized TR-EPR spectra of the lowest excited triplet states of **3**, **1**, **4**, and **2** in 2-MeTHF at 80 K.

This study establishes that integrating electron-poor phosphorus(V) porphyrin (or antimony(V) porphyrin) and electron-rich TPA units in a single molecular complex creates strongly absorbing black dyes. The large redox potential difference between the porphyrin and TPA units results in strong CT character in the lowest excited singlet and triplet CT states, and the decay between them is promoted by spin–orbit coupling.

## Supporting Information Available

Physical methods, synthesis details, ESI mass, NMR, electronic absorption, and electrochemistry data; solvent-dependent UV–visible absorption spectra, TD-DFT data, luminescence data, energy diagrams, spectroelectrochemistry data, transient absorption studies, and transient EPR studies (PDF)
