# Supplementary material for: Intramolecular Charge Transfer and Spin–Orbit Coupled Intersystem Crossing in Hypervalent Phosphorus(V) and Antimony(V) Porphyrin Black Dyes
Source: J Am Chem Soc. 2024 Sep 9;146(37):25403–8. doi: 10.1021/jacs.4c06674 (PMC11421002; doi:10.1021/jacs.4c06674)
Supplement: Supplementary file 1 — ja4c06674_si_001.pdf [file ja4c06674_si_001.pdf]

Supporting Information

Intramolecular Charge Transfer and Spin-Orbit Coupled Intersystem Crossing in Hypervalent Phosphorus(V) and Antimony(V) Porphyrin Black Dyes

Jam Riyan Hamza,[a] Jatan K. Sharma,[b] Paul A. Karr,[c] Art van der Est,[*] Francis D'Souza,[b,] Prashanth K. Poddutoori[a,*]*

[a]Department of Chemistry & Biochemistry, University of Minnesota Duluth, 1038 University Drive, Duluth, Minnesota 55812, USA.

[b]Department of Chemistry, University of North Texas, 1155 Union Circle, # 305070, Denton, Texas 76203-5017, USA.

[c]Department of Physical Sciences and Mathematics, Wayne State College, 1111 Main Street, Wayne, Nebraska 68787, USA.

[d]Department of Chemistry, Brock University, St. Catharines, ON L2S 3A1, Canada.

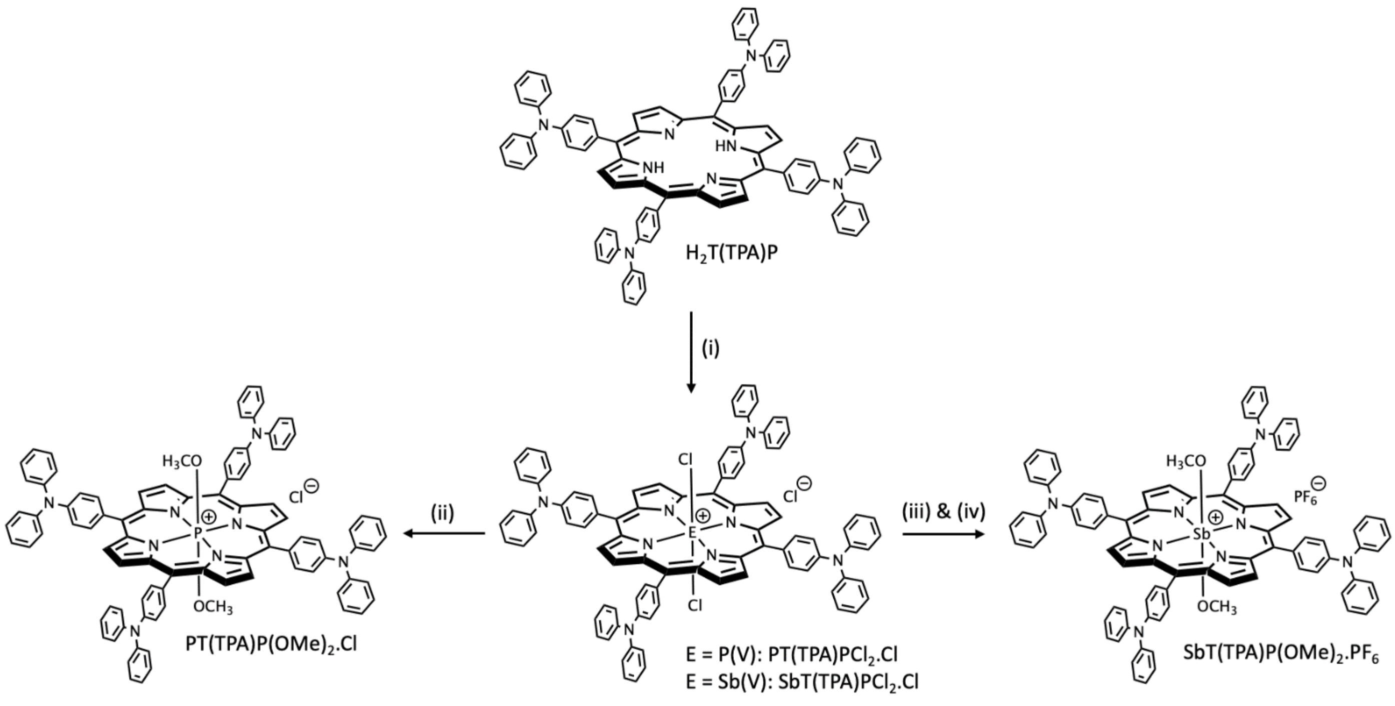

Scheme S1. Synthesis of the investigated compounds. *Reaction conditions*: (i) $POCl_3$ (or $SbCl_5$) in dry pyridine, N_2 (ii) dry CH_3OH, dry $CHCl_3$, dry pyridine, N_2 (iii) dry CH_3OH, dry pyridine, N_2, (iv) CH_3OH, aq. NH_4PF_6.

Experimental details

General. The chemicals and solvents utilized in this study were purchased from Alfa-Asear, Fisher Chemical, Acros Organics, Sigma-Aldrich, Tokyo Chemical Industry (TCI), or Accela and were used as received. Chromatographic materials were purchased from SiliCycle or Sigma-Aldrich. The synthesis of H_2T(TPA)P has been reported elsewhere.[1] The other compounds used in this study were prepared as described below.

Synthesis of [PT(TPA)PCl$_2$]Cl. A round bottom flask was charged with H_2T(TPA)P (104 mg, 0.081 mmol), a stirrer bar, a condenser, and an N_2 line. After flushing the flask with N_2 for 30 minutes, anhydrous pyridine (2 mL) was added using a syringe. After adding pyridine, $POCl_3$ (0.2 mL) was added quickly using a syringe, and the reaction mixture was refluxed under N_2 for 2-2.5 days. Pyridine was then removed using vacuum distillation. The product was purified using neutral Al_2O_3 column chromatography, where a CH_2Cl_2:CH_3OH (= 95:5) solvent mixture was used to elute the product on the column. The solvent was removed using rotatory evaporation, and 63mg of product was collected: Yield = 63 mg (55%). ^{1}H NMR ($CDCl_3$, 400 MHz): *δ, ppm* 9.14 (d, 8H, J = 4.5 Hz), 7.76 (d, 8H, J = 8.5 Hz), 7.33-7.40 (m, 40H), 7.18 (t, 8H, J = 7.3 Hz).

Synthesis of [SbT(TPA)PCl$_2$]Cl. A round bottom was charged with H_2T(TPA)P (100 mg, 0.078 mmol), a stirrer bar, a condenser, and an N_2 line. After flushing the flask with N_2 for 30 minutes, anhydrous pyridine (5 mL) was then added using a syringe. After adding pyridine, $SbCl_5$ (0.5 mL) was added drop by drop using a syringe, and the reaction mixture was refluxed under N_2 for 1-2 hours. Pyridine was then removed using vacuum distillation. The reaction mixture was re-dissolved in CH_2Cl_2:CH_3OH (85:15) mixture to stir overnight. The solution was then filtered through celite and dried under vacuum. The product was purified using basic Al_2O_3 column chromatography, where a CH_2Cl_2:CH_3OH (= 99:1) solvent mixture was used to elute the product on the column. The solvent was removed using rotatory evaporation, and the product was collected: Yield = 89 mg (76%). ^{1}H NMR ($CDCl_3$, 400 MHz): *δ, ppm* 9.74 (s, 8H), 8.14 (d, 8H, J = 8.2 Hz), 7.49-7.56 (m, 48H).

Synthesis of [PT(TPA)P(OMe)$_2$]Cl (1). [PT(TPA)PCl$_2$]Cl (50 mg, .035 mmol) was dissolved in anhydrous pyridine:CH_3OH (= 1:1) mixture, and the reaction mixture was refluxed under a nitrogen atmosphere for 7.5 hours. The reaction was then dried under vacuum and was purified using neutral Al_2O_3, where a CH_2Cl_2:CH_3OH (= 96:4) solvent mixture was employed to elute the product. The solvent was removed using rotatory evaporation, and the product was collected: Yield = 42 mg (85%). ^{1}H NMR ($CDCl_3$, 400 MHz): *δ, ppm* 9.12 (d, 8H, J = 2.8 Hz), 7.70 (d, 8H, J = 8.5), 7.33-7.39 (m, 40H), 7.17 (t, 8H, J = 7.2, 14.5), –1.81 (d, 6H, J = 25 Hz). ESI MS: *m/z* 1373.4495 for $[M - Cl^-]^+$, calculated 1373.5354 for $C_{94}H_{70}O_2N_8P^+$.

Synthesis of [SbT(TPA)P(OMe)$_2$]PF$_6$ (2). [SbT(TPA)PCl$_2$]Cl (40 mg, 0.026 mmol) was dissolved in a mixture of dry $CHCl_3$:CH_3OH:anhydrous pyridine (= 2:2:1) and was refluxed for 5-6 days under a nitrogen atmosphere. The reaction mixture was occasionally monitored using mass spectrometry to confirm the product was being formed in the reaction mixture over the course of the reaction. After confirming that the product had been formed, the reaction mixture was dried under vacuum and purified using column chromatography on neutral Al_2O_3 where CH_2Cl_2:CH_3OH (= 99:1) solvent mixture was used to elute the product on the column. The solvent was then removed under a vacuum. For conversion to PF_6 salt, the

product was dissolved in a minimum amount of methanol, and anhydrous NH_4PF_6 (100 mg) was then dissolved in the same solution. DI water (5x the amount of methanol) was added over the course of 30-40 minutes to precipitate the PF_6 salt of the product by vacuum filtration and was further dried via vacuum to yield 32mg of the product. Yield = 32 mg (85%). ^{1}H NMR ($CDCl_3$, 400 MHz): δ, ppm 9.68 (s, 8H), 8.14 (d, 8H, J = 8.4 Hz), 7.55 (d, 8H, J = 8.4 Hz), 7.47 (d, 32H, J = 4.3 Hz), 7.23 (m, 8H), –2.10 (s, 6H). ^{19}F NMR ($CDCl_3$, 377 MHz): δ, ppm –73.1 (d, 6F, J = 713 Hz). ^{31}P NMR ($CDCl_3$, 162 MHz): δ, ppm –144.5 (sept, J = 712.8). ESI MS: *m/z* 1463.3904 for $[M – PF_6]^+$, calculated 1463.4654 for $C_{94}H_{70}O_2N_8Sb^+$.

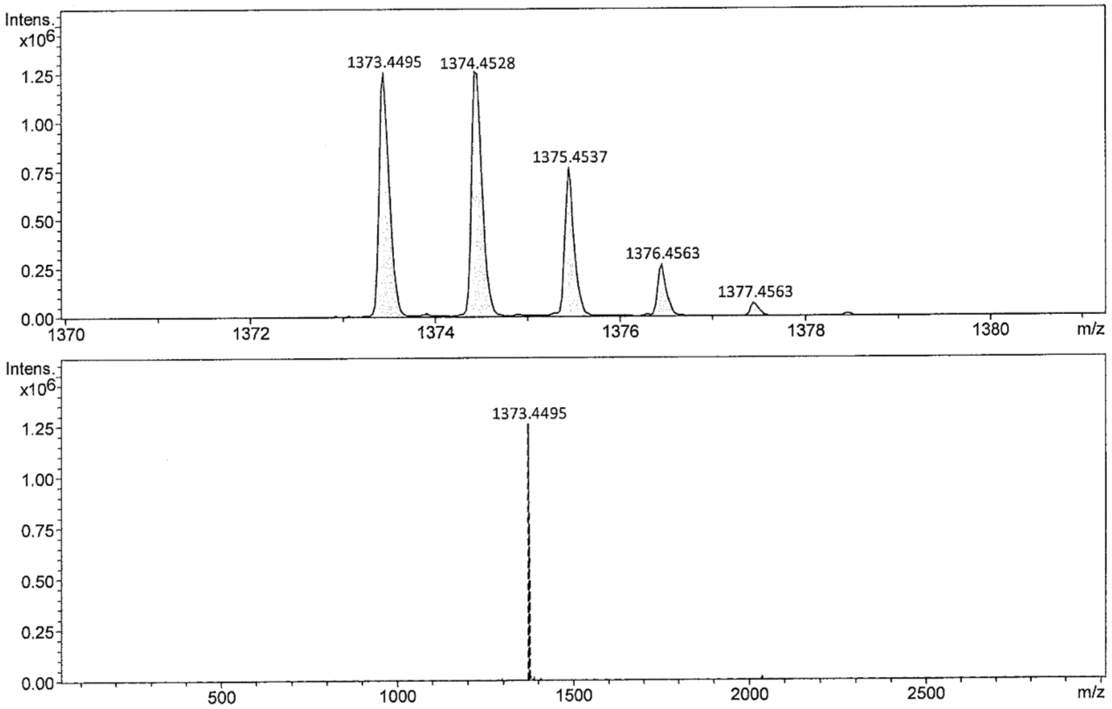

Figure S1. ESI mass spectrum of $[PT(TPA)P(OMe)_2]^+$ (**1**).

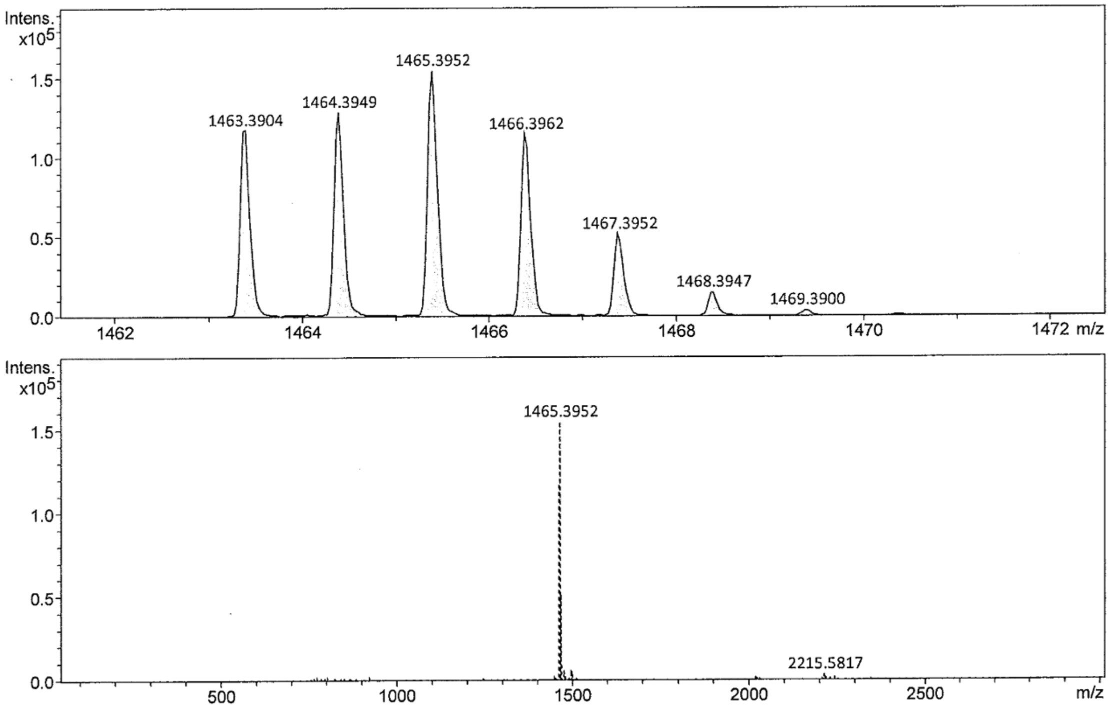

Figure S2. ESI mass spectrum of $[SbT(TPA)P(OMe)_2]^+$ (**2**)

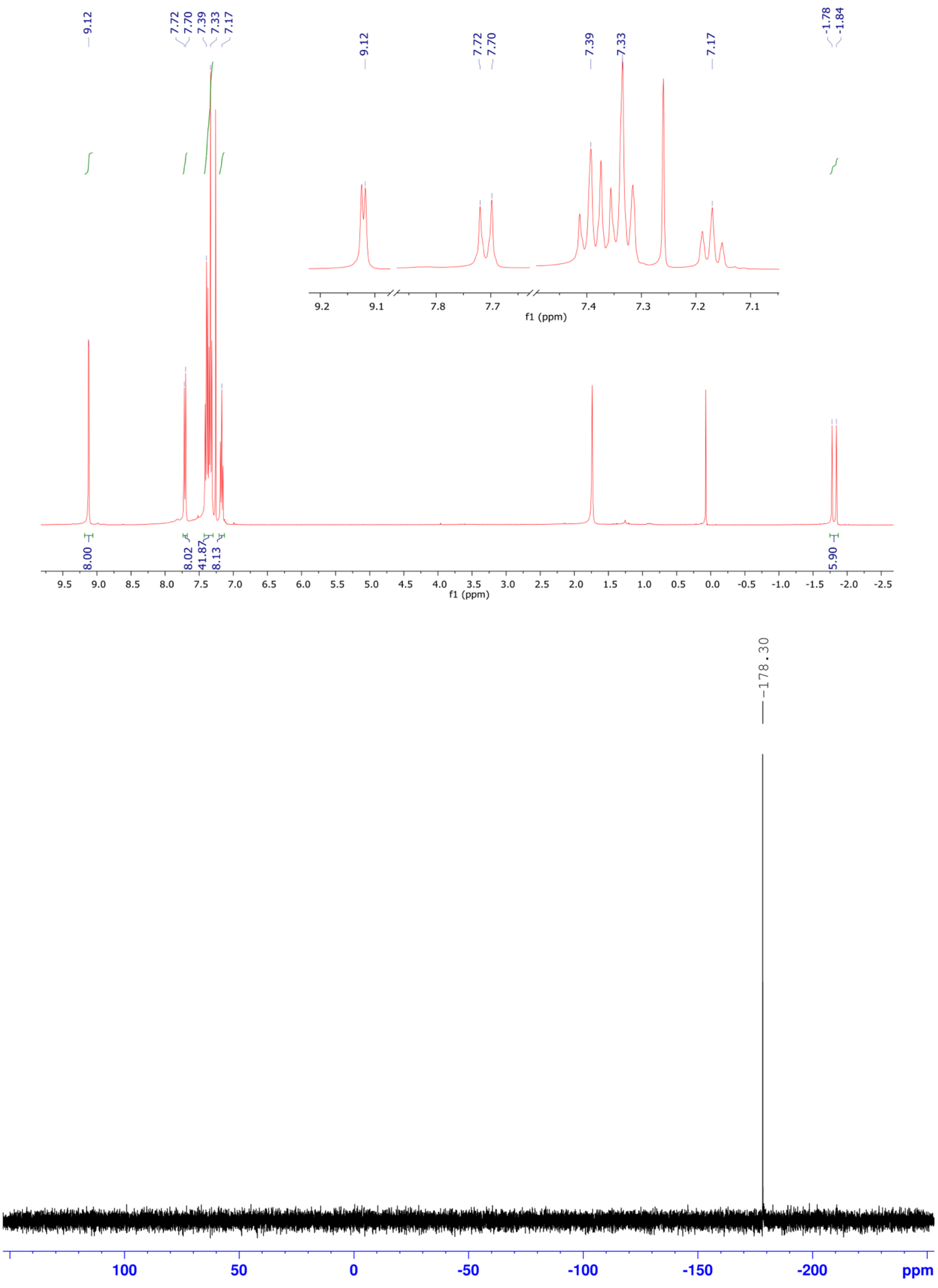

Figure S3. ^{1}H NMR (400 MHz, top) and ^{31}P NMR (162 MHz. bottom) spectra of [PT(TPA)P(OMe)$_2$]Cl (**1**)in $CDCl_3$.

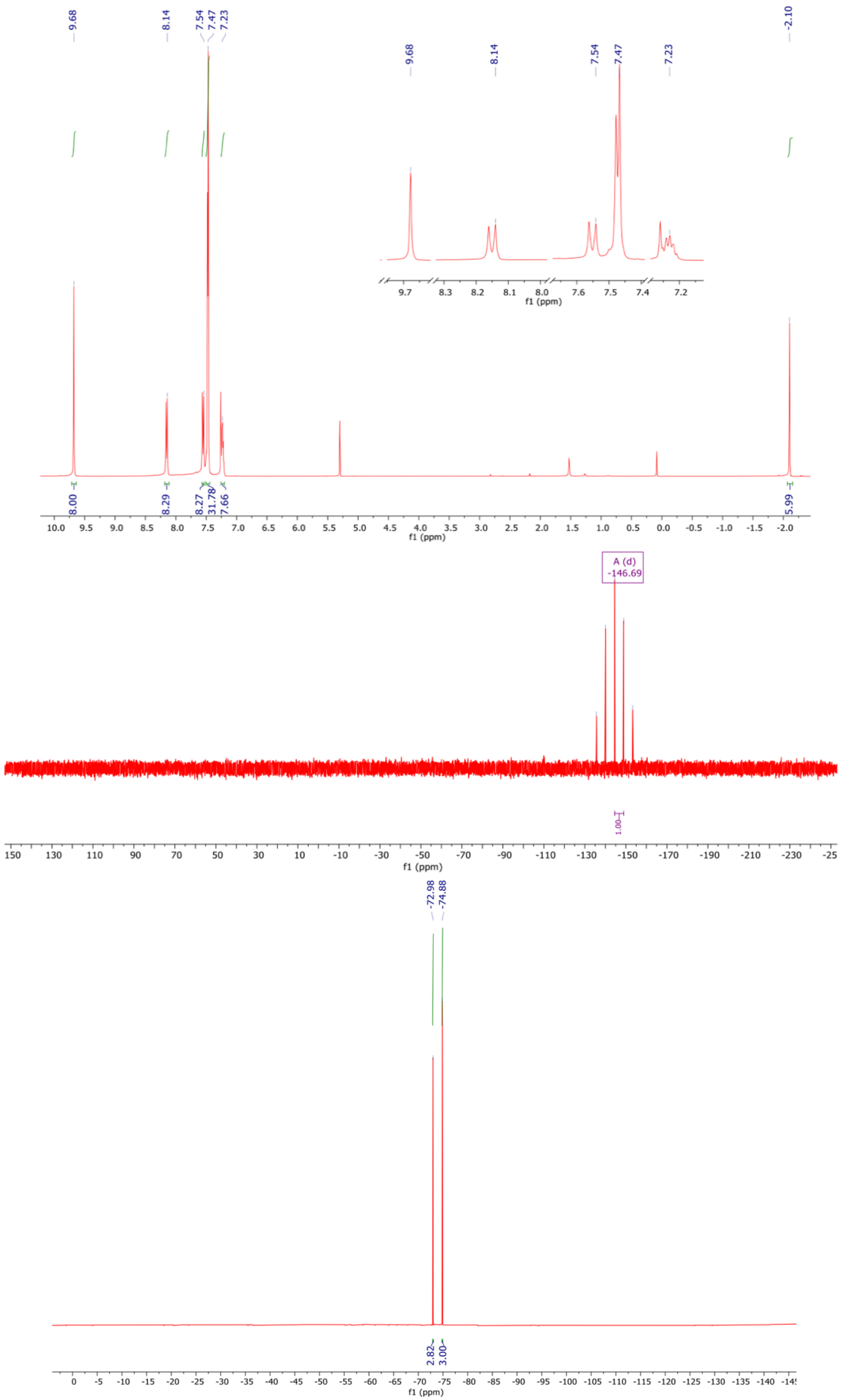

Figure S4. ^{1}H NMR (400 MHz, top), ^{31}P NMR (162 MHz, middle) and ^{19}F NMR (375 MHz, bottom) spectra of $[SbT(TPA)P(OMe)_2]PF_6$ (**2**) in $CDCl_3$.

Physical methods

NMR and mass spectroscopy. NMR spectra were recorded on a Bruker Advance 400 MHz NMR spectrometer using $CDCl_3$ as the solvent. ESI mass spectra were recorded on a Bruker MicroTOF-III mass spectrometer using direct injection from an UltiMate 3000 HPLC and acetonitrile as a solvent.

Absorption and emission spectroscopy. UV/Vis spectra were recorded with an Agilent Cary 100 UV/Vis spectrometer. The concentration of the samples used for these measurements ranged from 5×10^{-6} M (porphyrin B- band (Soret)) to 5×10^{-5} M (Q-bands) solutions. Steady-state fluorescence spectra were recorded using a Photon Technologies International Quanta Master 8075-11 spectrofluorometer, equipped with a 75 W Xenon lamp, running with FelixGX software. Emission spectra were collected using excitation in each of the major absorption bands. The samples were adjusted to 0.2 optical density at the selected excitation wavelength. Due to the low fluorescence quantum yields the excitation and emission slits were maintained at 3/3 nm. Constant sample concentration (4.5×10^{-6} M) was maintained in all CH_2Br_2 titrations.

Electrochemistry. Cyclic and differential pulse voltammetric experiments were performed on a BASi electrochemical analyzer (working electrode: Pt; auxiliary electrode: Pt wire; reference electrode: Ag wire). The Fc^+/Fc (Fc = ferrocene, $E_{1/2}(Fc^+/Fc) = 0.40$ V *vs*. SCE in CH_3CN, 0.1 M TBA·PF_6; $E_{1/2}(Fc^+/Fc) = 0.48$ V *vs*. SCE in CH_2Cl_2, 0.1 M TBA·ClO_4 under our experimental conditions) redox couple was used to calibrate the potentials.

DFT calculations. All the porphyrin structures were initially constructed on a local PC using the *GaussView 6* (GV6.0) software. DFT computations were performed on a supercomputer using the *Gaussian 16* software suite.[2] Since the investigation includes the theoretical study of the excited state and charge transfer properties where the highest excitation is to the LUMO+1, the B3LYP method was chosen. The 6-311+G(2df,pd) split-valence polarized basis set was used to model hydrogen and the period 2 elements (C, N, O) in the compounds. Since antimony is a period 5 element, the relativistic effects of the core electrons were modeled using effective core potentials (ECPs). The Stuttgart/Dresden ECPs, in combination with the triple-zeta polarized basis set (def2TZVPP), were chosen to model antimony for this study. Additionally, the Self-Consistent Reaction Field (SCRF) method, the Conductor Polarizable Continuum Model (CPCM), and the *Gaussian 16* parameters for acetonitrile were included to model the structures in solution. Thus, the B3LYP method was coupled with a GenECP basis to form the B3LYP/GenECP model chemistry, and SCRF(CPCM, Solvent=Acetonitrile) was used to optimize the geometry of all the structures herein to a stationary point on the Born-Oppenheimer surface and calculate the first ten excited singlet states of all chemical species in the current study. All the structures were optimized *sans* symmetry constraints as +1 charged cations and closed-shell singlets. The self-consistent field

(SCF) convergence constraints and the DFT grid utilized in the calculation were the G16 default values, "Tight" and "UltraFine" respectively. The optimization of the geometrical parameters of each of the chemical species in the study was continued until the maximum force, RMS force, maximum displacement, and RMS displacement reached or was less than the default *Gaussian 16* minima and the predicted energy change upon a successive optimization cycle of the geometrical parameters was in the range of -5×10^{-9} A.U. To generate the difference density maps shown in Fig. 1, additional TDDFT calculations were carried out using Orca (release 5.0.1).[3,4] The methods were the same as for the Gaussian calculations except that the Los Alamos valence double-zeta with Hay–Wadt ECPs were used.[5–7] ESP map color scale 4.0 – 8.0 × 10^{-2} V. The orbitals have been plotted with an isovalue of 0.02 and the DD maps at an isovalue of 0.0002.

Transient EPR spectroscopy. Transient EPR time/field data sets were recorded at 80 K using a modified Bruker EPR 200D-SRC X-band spectrometer equipped with a Flexline dielectric resonator and a CF 935 cryostat. A frequency-doubled Continuum Surelite Nd:YAG laser was used for pulsed light excitation at 532 nm with at a repetition rate of 10 Hz and 10 ns pulse width. The samples were prepared by dissolving each porphyrin in 200 μL of 2-methylTHF to a concentration of approximately 0.7 mM. The resulting solutions were degassed by several freeze-pump-thaw cycles. The samples were then frozen in a clear glass and placed in the cryostat. The spin-polarized TREPR spectra were extracted from the full-time/field dataset by taking the average signal intensity in a 500 ns time window centered at 750 ns after the laser flash and subtracting the average signal level before the laser flash. The spectra were simulated using EasySpin[8] is described below.

Femtosecond laser flash photolysis. Femtosecond transient absorption experiments were performed using an ultrafast femtosecond laser source (Libra) by Coherent incorporating a diode-pumped, mode-locked Ti:sapphire laser (Vitesse) and a diode-pumped intracavity doubled Nd:YLF laser (Evolution) to generate a compressed laser output of 1.45 W. Samples were excited at 410 nm. A Helios transient absorption spectrometer coupled with a femtosecond harmonics generator, provided by Ultrafast Systems LLC, was used for optical detection. The sources for the pump and probe pulses were derived from the fundamental output of Libra (Compressed output 1.45 W, pulse width 100 fs) at a repetition rate of 1 kHz; 95% of the fundamental output of the laser was introduced into a TOPAS-Prime-OPA system with a 290−2600 nm tuning range from Altos Photonics Inc., (Bozeman, MT), while the rest of the output was used for generation of a white light continuum. Kinetic traces at appropriate wavelengths were assembled from the time-resolved spectral data. Data analysis was performed using Surface Xplorer software supplied by Ultrafast Systems. All measurements were conducted in degassed solutions at 298 K. The estimated error in the reported rate constants is ±10%.

UV-Visible Absorption Data

Table S1. Absorption wavelengths and extinction coefficients of the investigated compounds in CH_3CN.

**Porphyrin**	**Absorption λ in nm (log ε in $M^{-1}cm^{-1}$)**
$[PTPP(OMe)_2]PF_6$ (**3**)	427 (4.53), 558 (3.23), 599 (2.63)
$[PT(TPA)P(OMe)_2]Cl$ (**1**)	299 (4.94), 409 (5.23), 496 (4.70), 570 (4.17), 653 (4.36)
$[SbTPP(OMe)_2]PF_6$ (**4**)	418 (6.01), 511 (3.91), 550 (4.61), 590 (4.40)
$[SbT(TPA)P(OMe)_2]PF_6$(**2**)	300 (4.94), 409 (5.64), 493 (4.56), 659 (4.49)
TPA	296 (5.05)

DFT Calculations

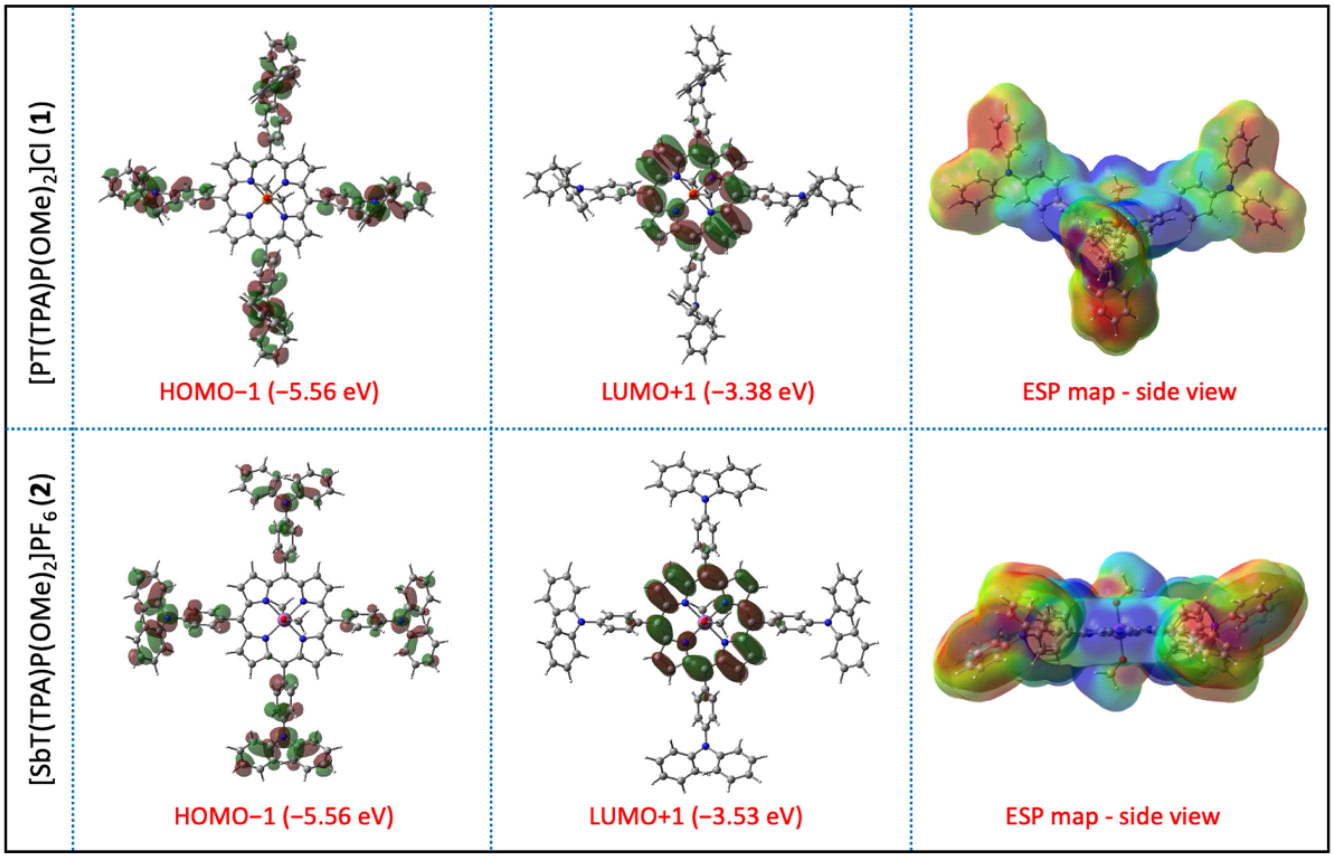

Figure S5. Calculated frontier orbitals and electrostatic potential maps (ESP, Blue = electron acceptor region. Red = electron donor region, color scale 4.000 – 8.000 × 10^{-2} V) of **1** and **2**.

Electrochemistry

To better comprehend the electrostatic potential differences, the electrochemical studies of **1** and **2** were performed in CH_3CN with 0.1 M TBA·PF_6, (see Figure S6), and Table S2. The cathodic scan reveals two one-electron reduction processes between –0.53 and –1.01 V for **1** and −0.37 and −0.84 V for **2,** corresponding to the successive addition of two electrons to the LUMO localized primarily on the porphyrin ring. The positively shifted potentials indicate these systems' strong electron acceptor nature. Moreover, the shift is greater in **2** compared to **1**, indicating that its porphyrin ring is even more electron deficient, making it a superior electron acceptor. The anodic scan reveals oxidation processes at 0.96 and 1.15 V for **1** and 1.04 V for **2,** corresponding to the removal of electrons from the HOMO. The current values for the oxidation are significantly higher than their corresponding reduction currents suggesting that the HOMO is localized on the TPA units. The absence of porphyrin-centered oxidation processes is consistent with the electron deficiency on the central ring.

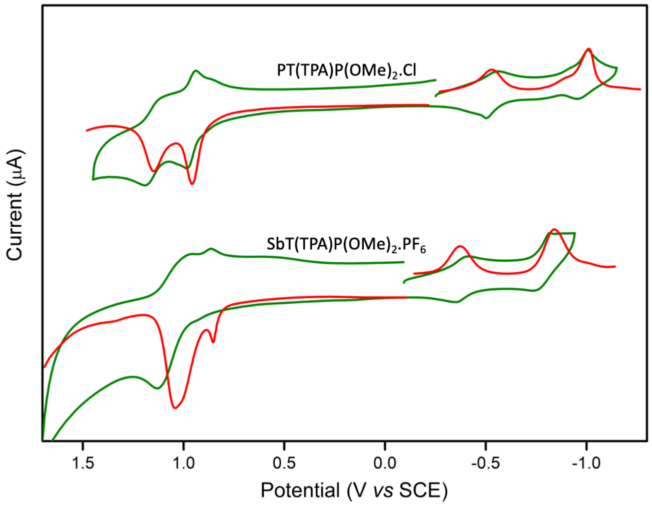

Figure S6. Cyclic and differential voltamograms of [PTTPAP(OMe)$_2$]Cl (**1**)and [SbTTPAP(OMe)$_2$]PF_6 (**2**) in CH_3CN with 0.1 M [TBA]PF_6. Scan rate 100 mV/s. Pulse period 200 ms, Pulse amplitude 50 mV, Pulse width 50 ms.

Table S2. Redox data of the investigated compounds in CH_3CN with 0.1 M [TBA]PF_6.

Sample	Potentials (V *vs.* SCE)	
	Reduction	Oxidation
[PTPP(OMe)$_2$]PF_6 (**3**)	−0.53, −0.98	1.65
[PT(TPA)P(OMe)$_2$]Cl (**1**)	−0.53, −1.01	0.96, 1.15
[SbTPP(OMe)$_2$]PF_6 (**4**)	–0.33, –0.75	1.78
[SbT(TPA)P(OMe)$_2$]PF_6 (**2**)	−0.37, −0.84	1.04
TPA	-	0.96

TD-DFT Studies

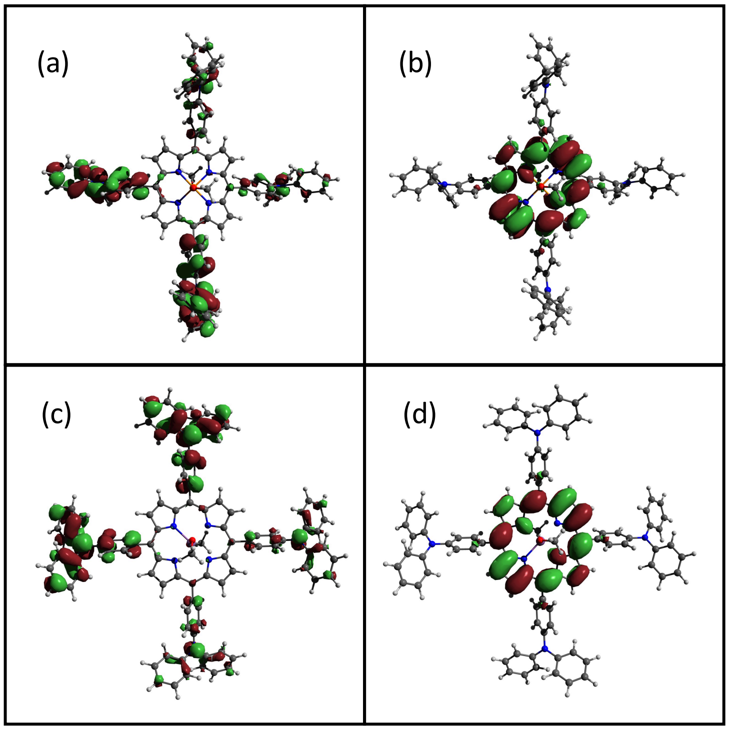

Figure S7. Natural transition orbitals (isovalue 0.02) of the lowest excited singlet states of **1** (panels a and b) and **2** (panels c and d). Panels a and c are the NTOs of the electron hole. Panels b and d are the NTOs of the excited electron.

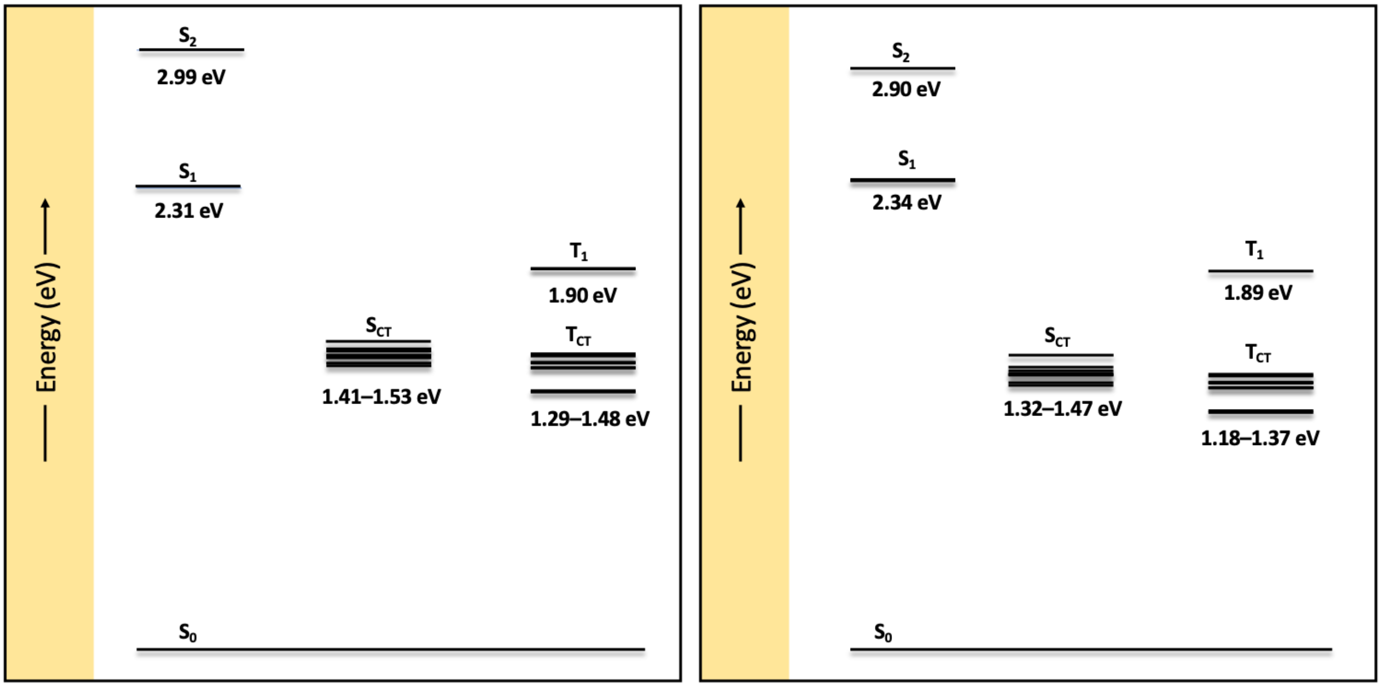

Figure S8. Energy level diagrams of **1** (left) and **2** (right) from TD-DFT studies.

Table S3. Absorption spectrum of **1** from TD-DFT.

ABSORPTION SPECTRUM VIA TRANSITION ELECTRIC DIPOLE MOMENTS

State	Energy (cm^{-1})	Wavelength (nm)	fosc	T^2 (au^2)	T_X (au)	T_Y (au)	T_Z (au)
1	11435.6	874.5	0.115208878	3.31668	1.81837	-0.00089	0.10107
2	11538.2	866.7	0.149023047	4.25196	0.00242	2.06203	-0.00010
3	11763.8	850.1	0.062239346	1.74178	-0.00232	-1.31976	-0.00060
4	11833.6	845.1	0.102726417	2.85786	-1.63571	0.00458	-0.42694
5	11853.4	843.6	0.043159905	1.19871	0.60649	0.00074	-0.91153
6	12016.8	832.2	0.010952162	0.30005	-0.00251	0.54776	0.00176
7	12111.2	825.7	0.039354591	1.06975	0.70910	-0.00003	-0.75295
8	12413.8	805.6	0.021643283	0.57398	-0.00047	-0.75761	-0.00124
9	18648.9	536.2	0.003366645	0.05943	0.24279	-0.00011	0.02204
10	18666.5	535.7	0.002088829	0.03684	0.00018	0.19194	-0.00001

Table S4. Absorption spectrum of **2** from TD-DFT.

ABSORPTION SPECTRUM VIA TRANSITION ELECTRIC DIPOLE MOMENTS

State	Energy (cm^{-1})	Wavelength (nm)	fosc	T^2 (au^2)	T_X (au)	T_Y (au)	T_Z (au)
1	10636.7	940.1	0.129597393	4.01111	2.00277	0.00012	0.00576
2	10749.1	930.3	0.121619990	3.72484	0.00017	-1.92998	-0.00000
3	11047.1	905.2	0.195128990	5.81500	0.00059	2.41143	0.00002
4	11069.9	903.3	0.083928940	2.49599	1.57819	-0.00088	0.07278
5	11126.9	898.7	0.078919422	2.33499	1.52806	-0.00004	-0.00402
6	11225.3	890.8	0.027231574	0.79864	0.00017	0.89367	0.00001
7	11366.2	879.8	0.062071001	1.79783	-1.33140	0.00001	-0.15879
8	11852.0	843.7	0.007145888	0.19849	0.00001	0.44552	0.00000
9	18885.3	529.5	0.010451210	0.18219	0.42683	0.00001	0.00026
10	18934.8	528.1	0.007667859	0.13332	0.00001	-0.36513	-0.00000

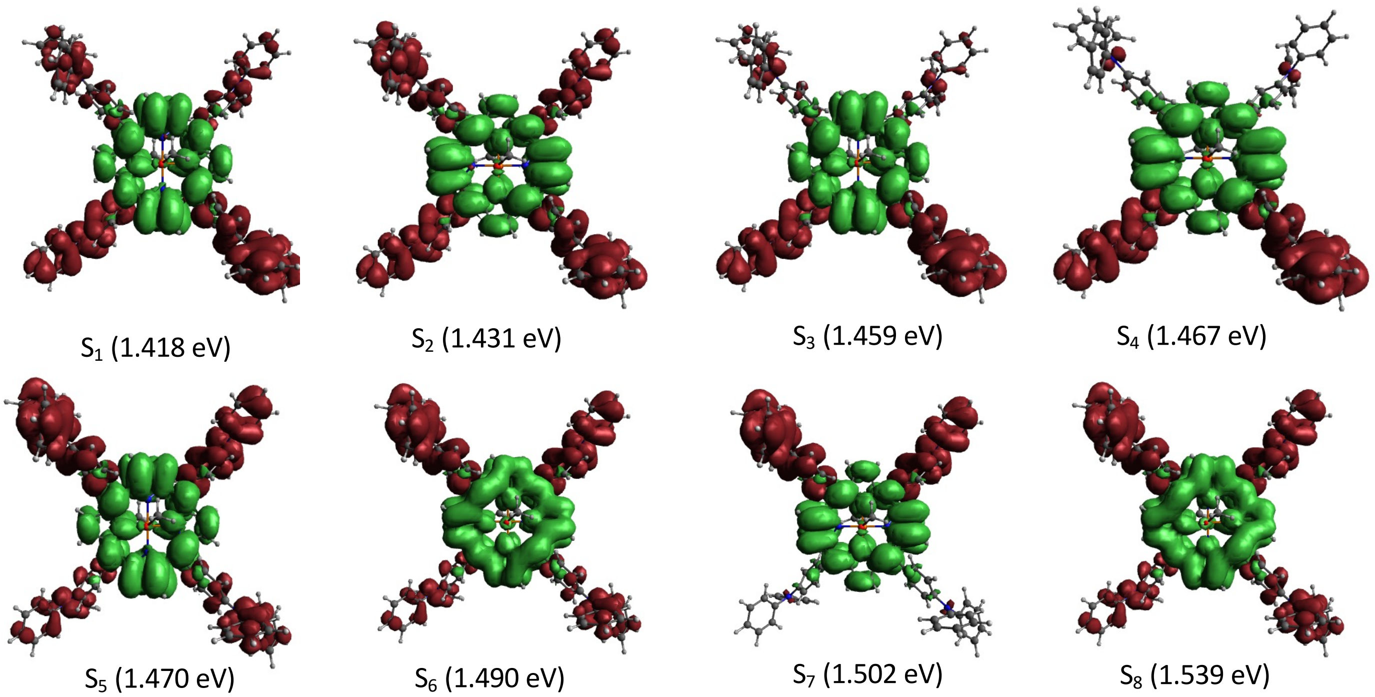

Figure S9. Density difference maps (isovalue 0.0002) of the eight lowest singlet states of **1**. The surfaces indicate the change in electron density in going from the ground state to the excited singlet state. Red: electron density decrease, green: electron density increase.

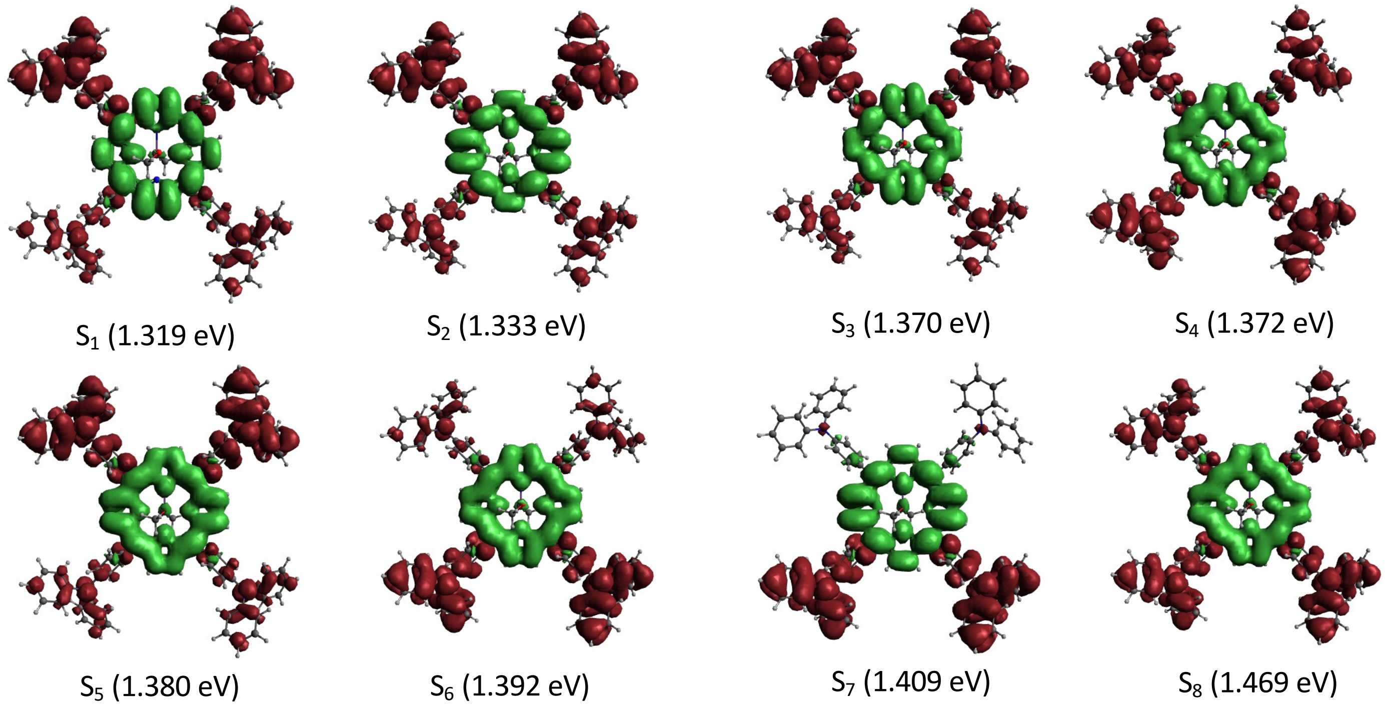

Figure S10. Density difference maps (isovalue 0.0002) of the eight lowest singlet states of **2**. The surfaces indicate the change in electron density in going from the ground state to the excited singlet state. Red: electron density decrease, green: electron density increase.

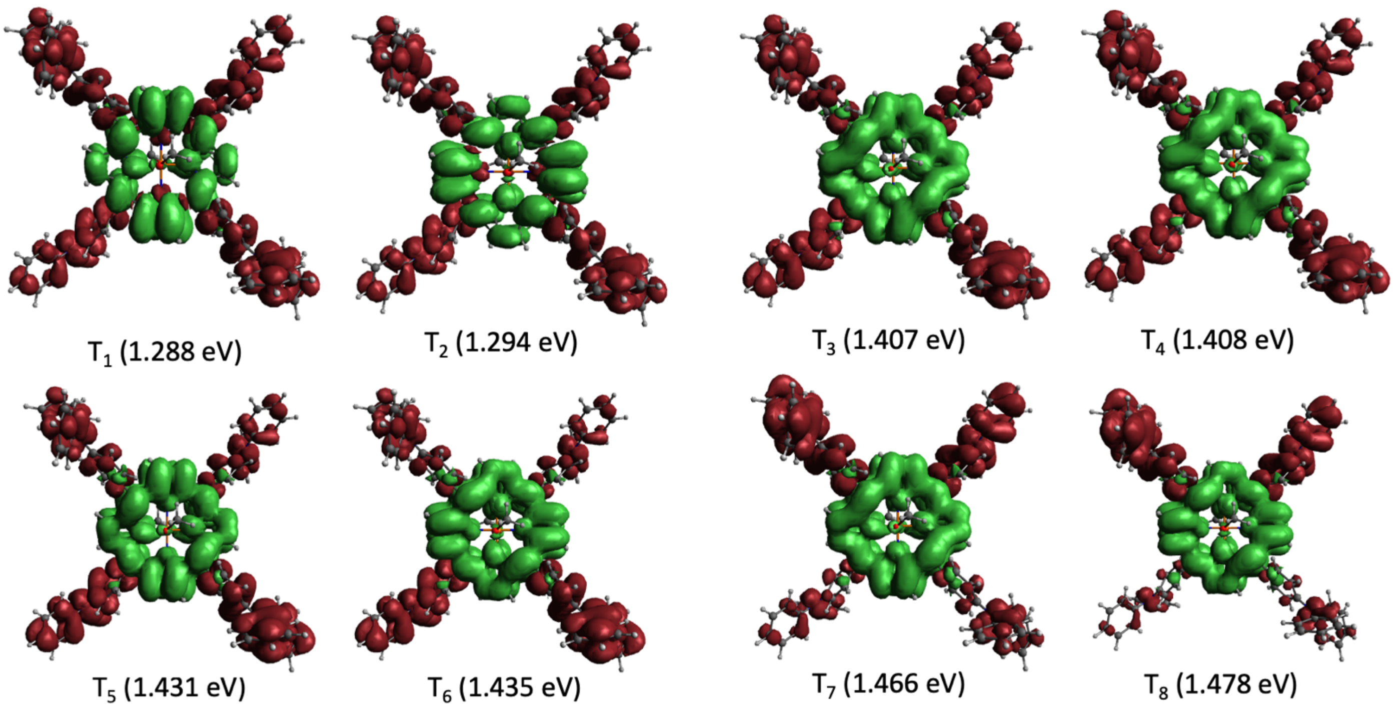

Figure S11. Density difference maps (isovalue 0.0002) of the eight lowest triplet states of **1**. The surfaces indicate the change in electron density in going from the ground state to the excited triplet state. Red: electron density decrease, green: electron density increase.

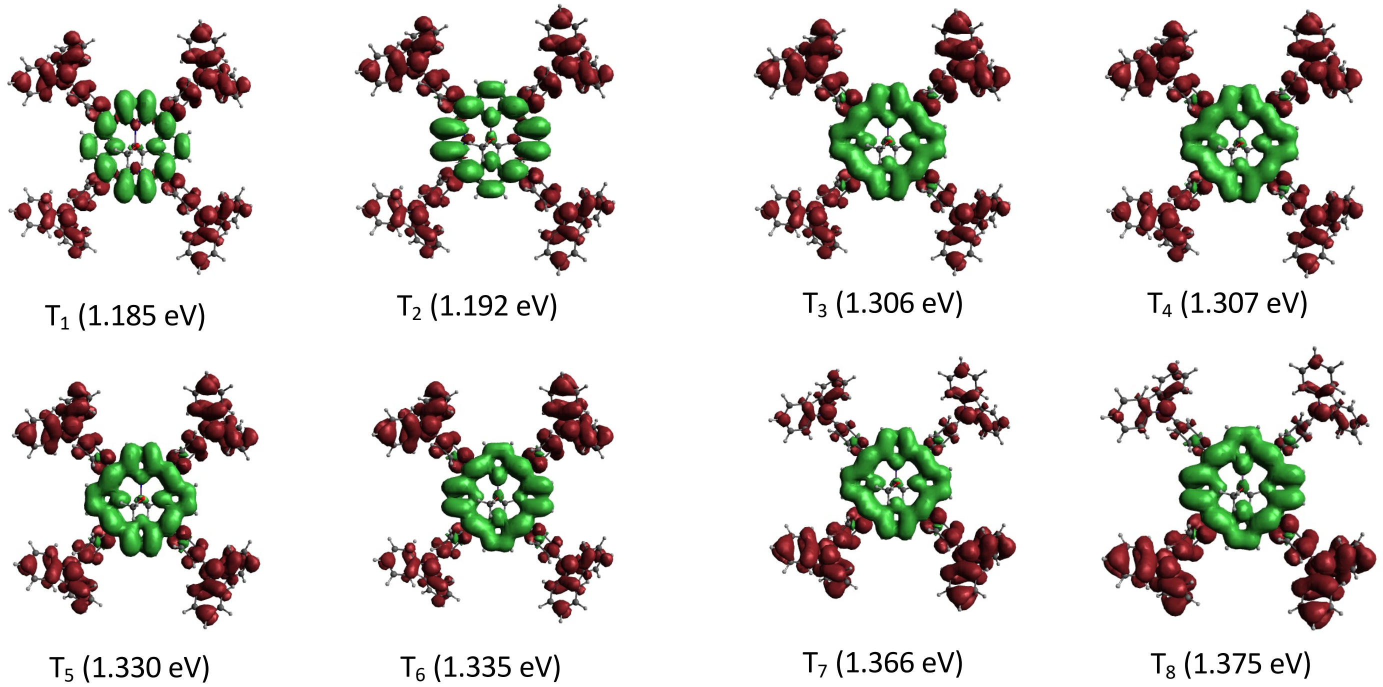

Figure S12. Density difference maps (isovalue 0.0002) of the eight lowest triplet states of **2**. The surfaces indicate the change in electron density in going from the ground state to the excited triplet state. Red: electron density decrease, green: electron density increase.

Solvent Dependent UV-Visible Absorption Studies

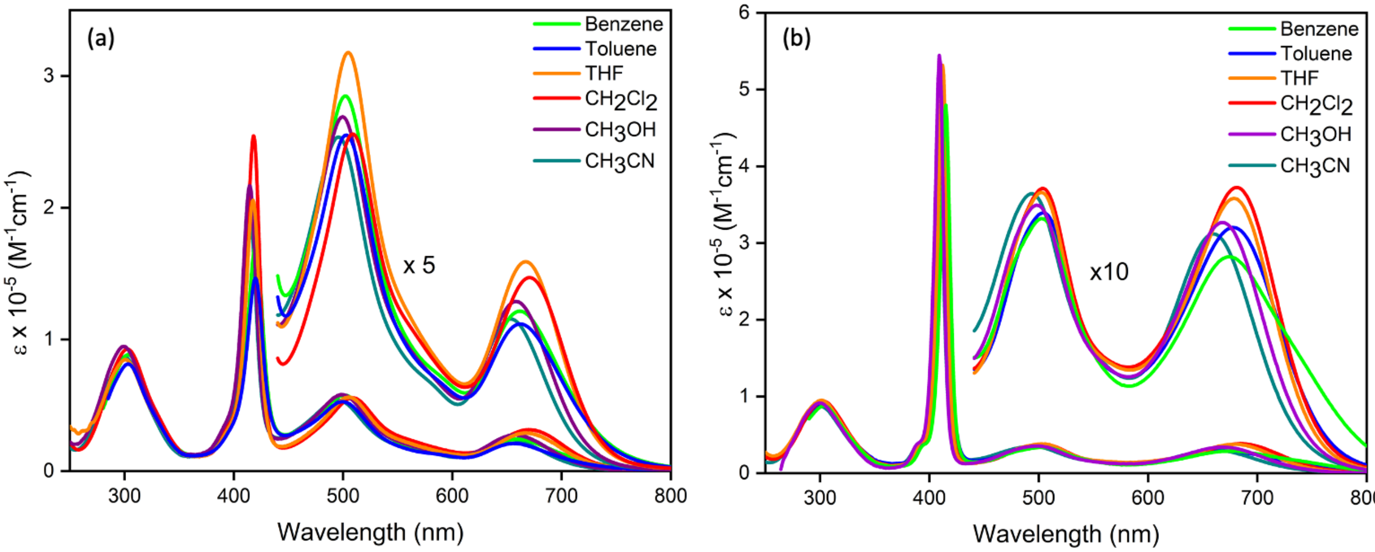

Figure S13. UV-visible absorption spectra of (a) $[PT(TPA)P(OMe)_2]Cl$ (**1**) and (b) $[SbT(TPA)P(OMe)_2]PF_6$ (**2**) in different solvents.

Luminescence Studies

The presence of ICT states leads to very strong quenching of the fluorescence with estimated quantum yields <0.01. Because of the quenching, reliable values of the quantum yields are difficult to obtain and, hence, are not reported.

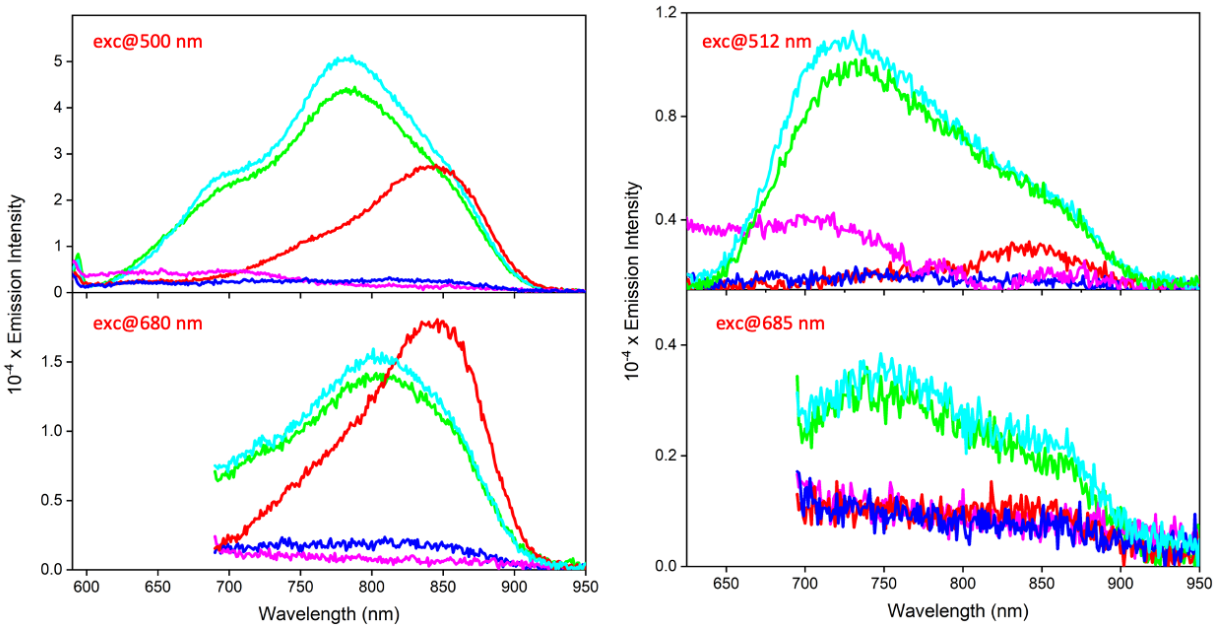

Figure S14. Fluorescence spectra of $[PT(TPA)P(OMe)_2]Cl$ (**1**, left) and $[SbT(TPA)P(OMe)_2]PF_6$ (**2**, right) in benzene (green), toluene (cyan), CH_2Cl_2 (red), CH_3CN (magenta) and CH_3OH (blue) at room temperature. Excitation wavelengths are given in the image.

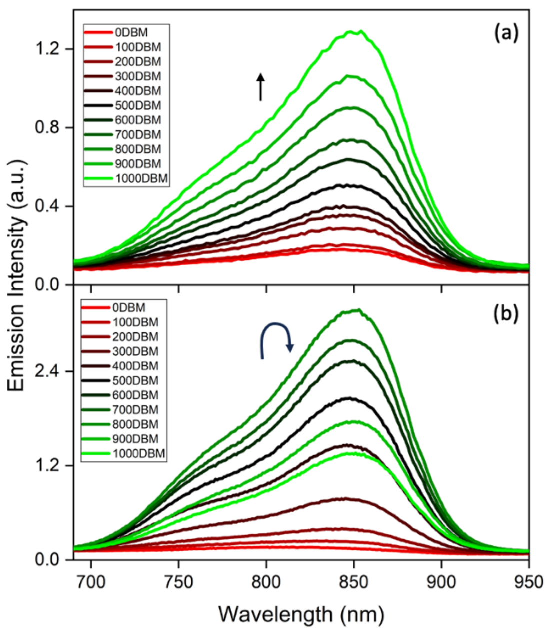

Figure S15. Emission spectra of $[PT(TPA)P(OMe)_2]Cl$ (**1**) with increasing volumes (0 to 1000 μL in increments of 100 μL) of CH_2Br_2 at 680 nm excitation in (a) CH_2Cl_2 and (b) toluene. Constant sample concentration (4.9×10^{-6} M) was maintained in all titrations.

The CH_2Br_2 titrations were also carried out on **2**, and similar results were obtained (Figure S16), except that the emission intensities are an order of magnitude lower than for **1**.

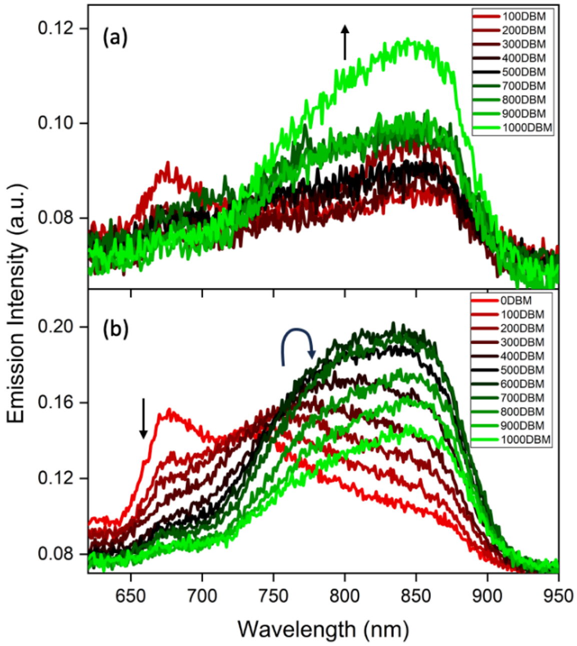

Figure S16. Emission spectra of $[SbT(TPA)P(OMe)_2]PF_6$ (**2**) with increasing volumes (0 to 1000 μL in increments of 100 μL) of CH_2Br_2 at 512 nm excitation in (a) CH_2Cl_2, sample concentration = 5.1×10^{-6} M and (b) toluene, sample concentration = 9.6×10^{-6} M.

Triplet Quencher and Thermally Activated Delay Fluorescence (TADF) Studies

In order to understand the nature of the 850 nm emission band, triplet quenching experiments were performed using compound **1** in toluene, see Figure S17a. The emission was collected in the presence and absence of triplet quencher O_2 with excitation at 500 nm. The samples were purged with O_2 and N_2 gases for 30 min before collecting the emission. It is anticipated the proposed ^3CT state would undergo energy transfer with $^3O_2$ to produce $^1O_2$ with an energy of 0.97 eV.[1] Based on the energetics (Figure 3) the proposed energy transfer process is thermodynamically favorable under these conditions, and the intensity of the 850 nm band should decrease in the presence of O_2 gas. However, as shown in Figure S17a, the emission intensities are not very different with and without O_2 gas. These inconclusive results prompted us to attempt the same studies with ferrocene (Fc), see Figure S17b. Ferrocene's lowest energy triplet state is around 1.16 eV,[9] therefore, a triplet-triplet energy transfer from ^3CT to ^3Fc is thermodynamically feasible. In Figure S17b, it is evident that the emission intensity remains relatively stable even in the presence of a large excess. Overall, the results from experiments involving triplet quenchers are inconclusive. This may be due to these triplet quenchers reacting with the dark triplet states of the porphyrins, or the singlet/triplet states being mixed and not as susceptible to being quenched by the chosen triplet quenchers.

Temperature-dependent emission studies were performed in CH_2Cl_2 (Figure S17c) and in toluene (Figure S17d) to examine the thermally activated delay fluorescence (TADF). Compound **1** was excited at 500 nm and the emission was collected while increasing the temperature. If TADF is present in the molecule, the emission intensity would decrease. However, in CH_2Cl_2 solvent the mission intensity slightly increases whereas in toluene marginally decreases. Once again, the observed trends do not confirm the TADF mechanism in compound **1**.

The TCSPC fluorescence lifetime measurements at various emission wavelengths were carried out in toluene, see Table S5. These lifetimes are in the ns range, and as such, they do not show clearly whether the emission arises from the singlet or triplet state.

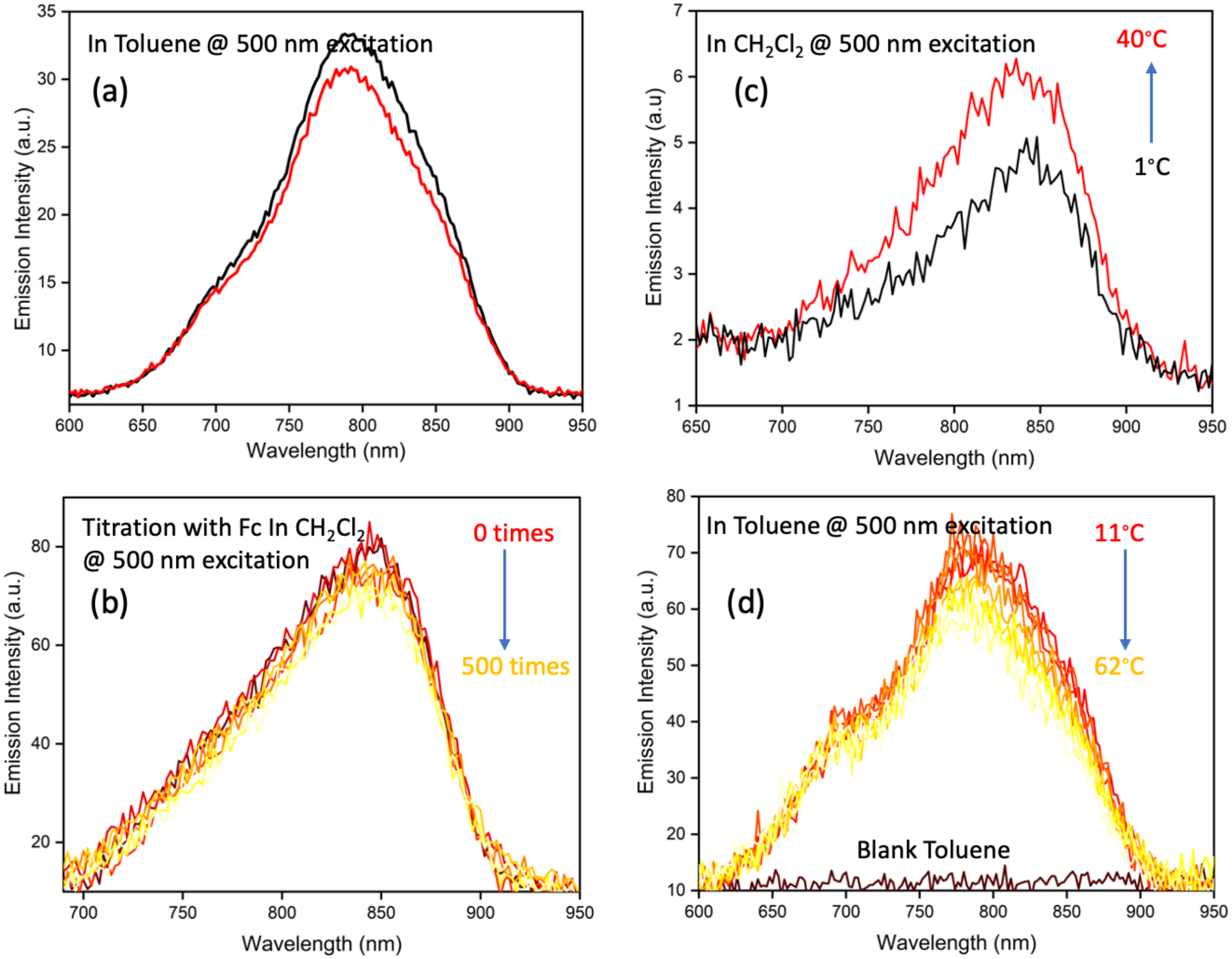

Figure S17. (a) Emission spectra of [PT(TPA)P(OMe)$_2$]Cl (**1**) in the presence of O_2 (red) and N_2 (black) gas. (b) Emission spectra of [PT(TPA)P(OMe)$_2$]Cl (**1**) in the presence of various amounts of ferrocene (Fc). Variable temperature emission spectra of [PT(TPA)P(OMe)$_2$]Cl (**1**) in (c) CH_2Cl_2, and (d) Toluene.

Table S5. TCSPC lifetime measurements of **1** in toluene at different temperatures. The sample was excited at 494 nm, and emission was collected at various wavelengths.

**Sample: 1**	**CHISQ**	**Avg. Lifetime (ns)**
Em650nm@5°C	1.19	1.55
Em700nm@5°C	1.19	1.20
Em775nm@5°C	1.19	1.06
Em850nm@5°C	1.18	0.86
Em650nm@RT	1.08	1.68
Em700nm@RT	1.17	0.72
Em775nm@RT	1.16	0.94
Em850nm@RT	1.17	0.89
Em650nm@70°C	1.17	1.85
Em700nm@70°C	1.20	1.11
Em775nm@70°C	1.20	0.92
Em850nm@70°C	1.14	0.95

Energetics

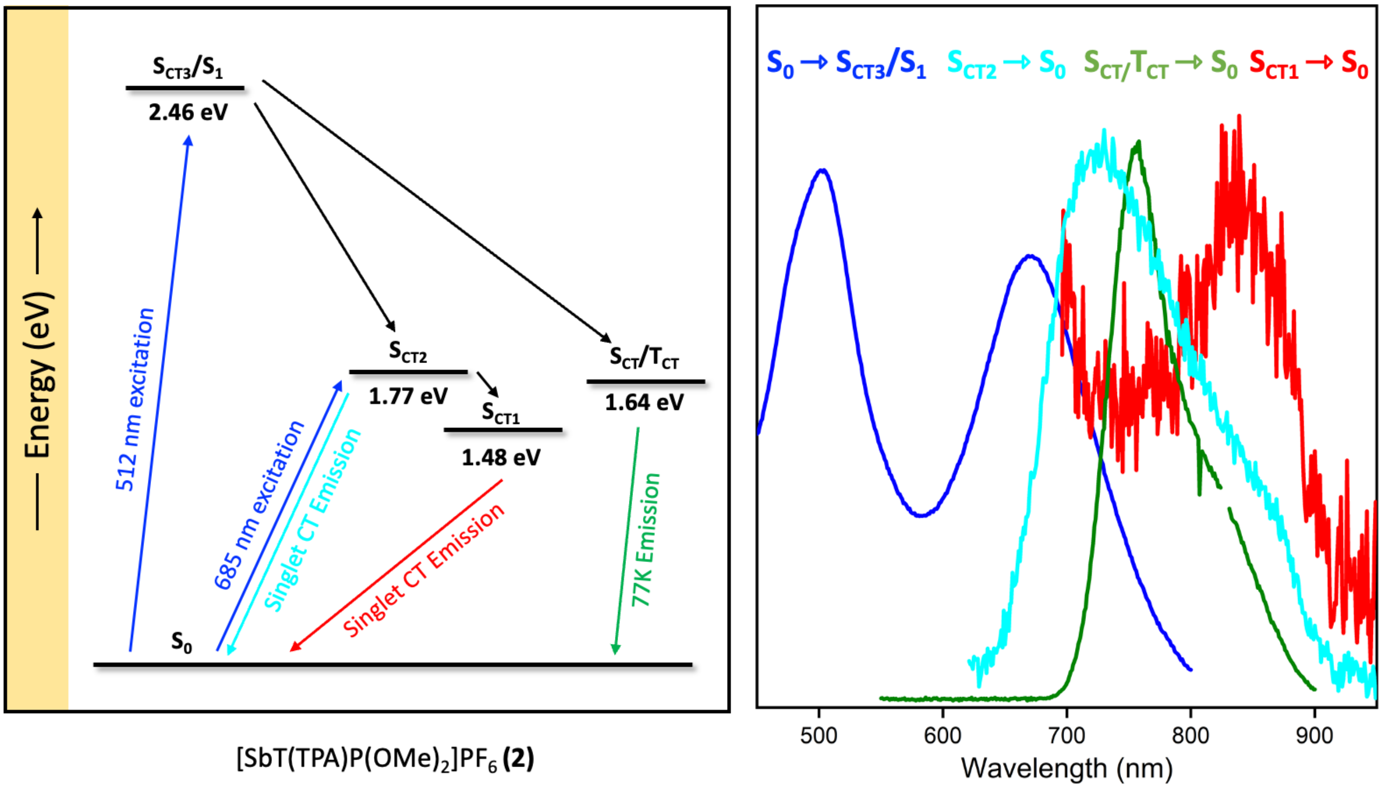

Figure S18. Left: Calculated energy level diagram of **2**. Right: Normalized spectral overlap of absorption in toluene (blue), emission in toluene (cyan) in CH_2Cl_2 (red) of **2** at ambient temperature. The green trace is the emission of **2** in a 2Me-THF:CH_2Cl_2 :1,2-dibromoethane (= 66:33:1) glass at 77K.

Spectroelectrochemistry Studies

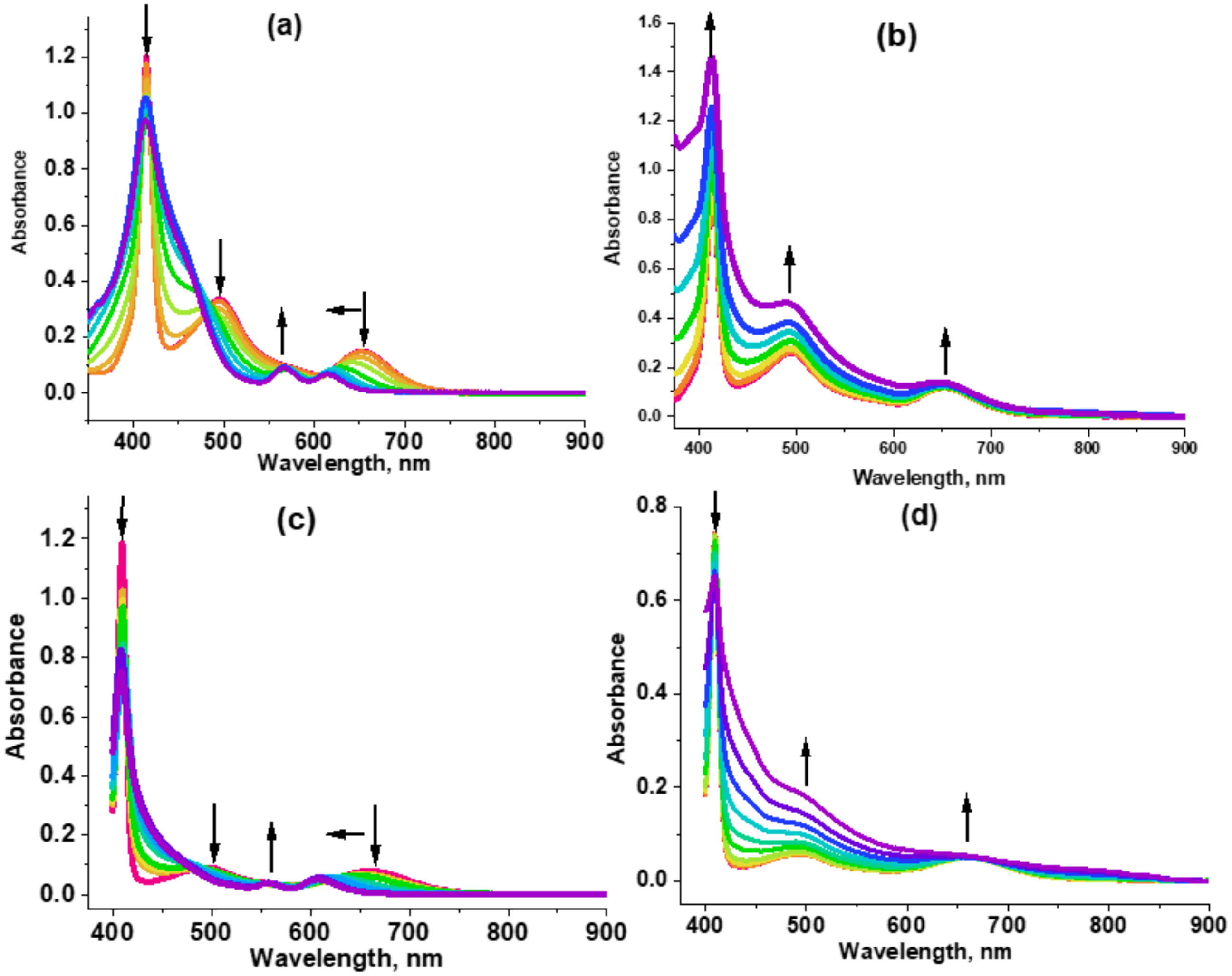

Figure S19. Spectral changes observed during chemical oxidation of (a) **1** and (c) **2**; and reduction of (b) **1** and (d) **2** in acetonitrile. Nitrosonium tetrafluoroborate and cobaltocene were used as oxidizing and reducing agents.

Fs-Transient Absorption Studies

Changing the solvent from polar CH_3CN to nonpolar toluene revealed appreciable changes in photodynamics, as shown in Figure S20. The expected ESA and GSB peaks complimenting the absorption peak maxima were also observed. For **1**, the DAS revealed four components, and from spectral comparison, the first spectrum at $\tau_1 = 3.9$ ps is attributed to the S_2 state, the second one at $\tau_2 = 42.2$ ps to the S_1 state, and the third one at $\tau_3 = 94.8$ ps to the charge transfer state (Figure S20a). A long-lived component at $\tau_4 > 3$ ns was also observed with features of the triplet excited state. From the initial growth and decay of the population time profiles (Figure S20b and c), the following sequence of occurrence: $S_2 \rightarrow S_1 \rightarrow$ CT $\rightarrow T_1$ could be suggested; however, looking at the initial time of growth, the S_1 state to simultaneously populate both CT and T_1 state cannot be ruled out. In the case of **2**, a four-component fit also provided satisfactory results (Figure S20d). In this case, the spectra at $\tau_1 = 3.6$ ps to the S_2 state, $\tau_2 = 26.3$ ps to the S_1 state, and $\tau_3 = 161.9$ ps for the CT state and $\tau_4 > 3$ ns to the triplet state were possible to assign (Figure S20e). From the initial growth and decay of the population time profiles (Figure S20f), the following simultaneous sequence of occurrence: $S_2 \rightarrow S_1 \rightarrow$ CT and $S_2 \rightarrow S_1 \rightarrow T_1$ was possible to envision.

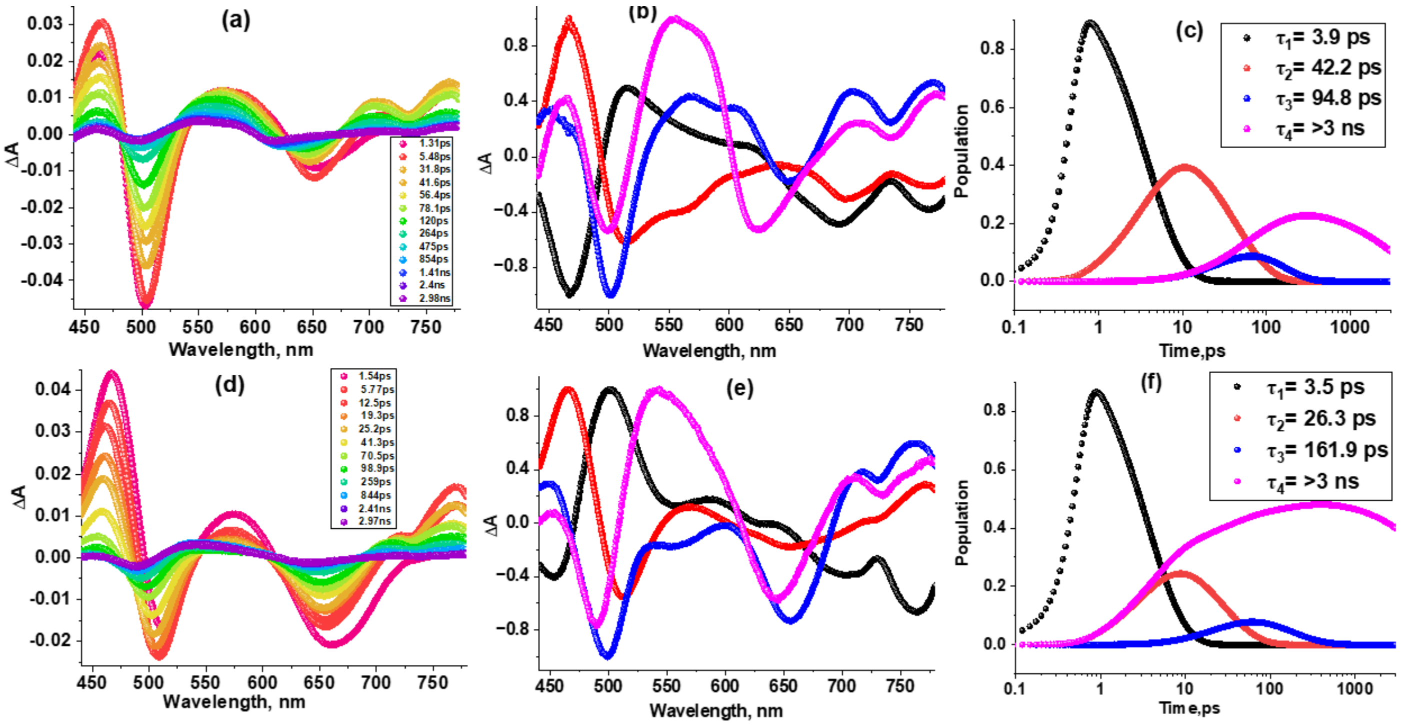

Figure S20. *Fs*-TA spectra at the indicated delay times of (a) **1** and (d) **2** in toluene at the excitation wavelength of 410 nm. Their corresponding decay-associated spectra (DAS) (b) and (e) and the population time profiles (c) and (f) are on the right.

Transient EPR Spectral Simulations

The spectra in Figure 5 have been normalized to similar maximum amplitudes and a microwave frequency of 9.8 GHz. The transient EPR spectra were simulated using EasySpin. Initial attempts to reproduce the experimental spectra using the EasySpin 5.2.35 function "pepper" did not reproduce important aspects of the experimental spectra. The origin of this issue is likely the dynamic Jahn-Teller Effect that is known to cause broadening of the spectral features from the x and y canonical orientations in metalloporphyrin triplet state spectra.[10] A full treatment of this effect requires a dynamic model involving back-and-forth transitions between two closely spaced triplet states. A simpler but less rigorous approach is to approximate the effect on the spectrum by including an orientation-dependent linewidth using the HStrain and DStrain parameters in EasySpin. A second problem is the orientation-dependent net polarization that is apparent in the spectrum. This polarization can be generated by several different possible mechanisms[11,12] but cannot be calculated directly using the function "pepper" in EasySpin. Thus, an additional routine was written to calculate its contribution. The spectra of individual orientations were calculated at thermal equilibrium using EasySpin and were then weighted with an orientation-dependent parameter described by vector. The individual orientation spectra were then summed to give the powder spectrum of the net polarization contribution, which was then added to a multiplet pattern obtained using "pepper".

Table S6. Zero-field splitting parameters and triplet sublevel population rates.

**Sample**	**Zero-field splitting parameters (MHz)**				**Relative triplet sublevel population rates**
	**Experiment**		**Calculated**		$(p_x - p_y) : (p_y - p_z)$
	**|D|**	**|E|**	**|D|**	**|E|**	
**3**	732	214	974	8.9	0.02 : 0.49
**1**	527	152	477	50	0.02 : 0.49
**4**	1005	337	1251	12.5	0.63 : –0.37
**2**	612	200	653	34.5	0.43 : –0.57

Table S7. Parameters used to simulate the TREPR spectra of compounds **4** and **2**.

**4**	
g-value	2.0029
D, E (MHz)	[1005, 337]
DStrain (MHz)	[0, 20]
Gaussian Linewidth (mT)	1.0
HStrain (MHz)	[0, 76, 110]
ZFS sublevel populations [p_x, p_y, p_z]	[0.63, 0, 0.37]
Net polarization	[-6.0×10^{-4}, 1.4×10^{-3}, 6.0×10^{-4}]

**2**	
g-value	2.0029
D, E (MHz)	[612, 200]
DStrain (MHz)	[0, 20]
Gaussian Linewidth (mT)	1.0
HStrain (MHz)	[0, 120, 150]
ZFS sublevel populations [p_x, p_y, p_z]	[0.43, 0, 0.57]
Net polarization	[-1.2×10^{-4}, 4.8×10^{-3}, 3.5×10^{-4}]

Table S8. Parameters used to simulate the TREPR spectra of compounds **3** and **1**.

**3**	
g-value	2.0023
D, E (MHz)	[732, 214]
DStrain (MHz)	[0, 126]
Gaussian Linewidth (mT)	1.0
HStrain (MHz)	[150, 100, 52]
ZFS sublevel populations [p_x, p_y, p_z]	[0.49, 0.51, 0]
Net polarization	[3.1×10^{-4}, -8.0×10^{-3}, -6.0×10^{-4}]

**1**	
g-value	2.0023
D, E (MHz)	[527, 152]
DStrain (MHz)	[0, 101]
Gaussian Linewidth (mT)	1.0
HStrain (MHz)	[150, 160, 68]
ZFS sublevel populations [p_x, p_y, p_z]	[0.49, 0.51, 0]
Net polarization	[1.1×10^{-4}, 6.8×10^{-6}, -7.0×10^{-4}]

The emission/absorption (E/A) pattern observed for **1** and **3** corresponds to the selective population of the T_x and T_y triplet sublevels at zero-field (Table S6). This pattern is expected in planar aromatic molecules because the orbital angular momentum of the lowest excited states lies in the xy plane. Mixing these states leads to a change in the orientation of the orbital angular momentum in this plane. In contrast, the sign of the polarization in **2** and **4** is A/E due to an out-of-plane rotation of the orbital angular. This difference is likely due to a larger contribution from the heavy Sb center to the orbital angular momentum and/or differences in the symmetries and frequencies of the vibrational modes of the vibronic states involved.

Reference

(1) Subedi, D. R.; Reid, R.; D'Souza, P. F.; Nesterov, V. N.; D'Souza, F. Singlet Oxygen Generation in Peripherally Modified Platinum and Palladium Porphyrins: Effect of Triplet Excited State Lifetimes and Meso-Substituents on 1O2 Quantum Yields. *Chempluschem* **2022**, *87* (4), 1–10.

(2) Frisch, M. J.; Trucks, G. W.; Schlegel, H. B.; Scuseria, G. E.; Robb, M. a.; Cheeseman, J. R.; Montgomery, J. a.; Vreven, T.; Kudin, K. N.; Burant, J. C.; Millam, J. M.; Iyengar, S. S.; Tomasi, J.; Barone, V.; Mennucci, B.; Cossi, M.; Scalmani, G.; Rega, N.; Petersson, G. a.; Nakatsuji, H.; Hada, M.; Ehara, M.; Toyota, K.; Fukuda, R.; Hasegawa, J.; Ishida, H.; Nakajima, T.; Honda, Y.; Kitao, O.; Nakai, H.; Klene, M.; Li, X.; Knox, J. E.; Hratchian, H. P.; Cross, J. B.; Adamo, C.; Jaramillo, J.; Gomperts, R.; Stratmann, R. E.; Yazyev, O.; Austin, A. J.; Cammi, R.; Pomelli, C.; Ochterski, J.; Ayala, P. Y.; Morokuma, K.; Voth, G. a.; Salvador, P.; Dannenberg, J. J.; Zakrzewski, V. G.; Dapprich, S.; Daniels, A. D.; Strain, M. C.; Farkas, O.; Malick, D. K.; Rabuck, A. D.; Raghavachari, K.; Foresman, J. B.; Ortiz, J. V.; Cui, Q.; Baboul, A. G.; Clifford, S.; Cioslowski, J.; Stefanov, B. B.; Liu, G.; Liashenko, A.; Piskorz, P.; Komaromi, I.; Martin, R. L.; Fox, D. J.; Keith, T.; Al-Laham, M. a.; Peng, C. Y.; Nanayakkara, A.; Challacombe, M.; Gill, P. M. W.; Johnson, B.; Chen, W.; Wong, M. W.; Gonzalez, C.; Pople, J. a. Gaussian 16. Gaussian Inc.: Wallingford, CT 2016.

(3) Neese, F. Wiley Interdisciplinary Reviews: *Computational Molecular Science* **2012**, *2*, 73–78.

(4) Nesse, F. Wiley Interdisciplinary Reviews: *Computational Molecular Science* **2018**, *8*, e1327.

(5) Hay, P. J. ; W. W. R. Ab Initio Effective Core Potentials for Molecular Calculations. Potentials for the Transition Metal Atoms Sc to Hg. *J. Chem. Phys.* **1985**, *82*, 270–283.

(6) Willard R. Wadt; P. Jeffrey Hay. Ab Initio Effective Core Potentials for Molecular Calculations. Potentials for Main Group Elements Na to Bi. *J. Chem. Phys.* **1985**, *82*, 284–298.

(7) P. Jeffrey Hay; Willard R. Wadt. Ab Initio Effective Core Potentials for Molecular Calculations. Potentials for K to Au Including the Outermost Core Orbitals. *J. Chem. Phys.* **1985**, *82*, 299–310.

(8) Stoll, S.; Schweiger, A. EasySpin, a Comprehensive Software Package for Spectral Simulation and Analysis in EPR. *Journal of Magnetic Resonance* **2006**, *178* (1), 42–55.

(9) Poddutoori, P. K.; Sandanayaka, A. S. D.; Hasobe, T.; Ito, O.; Van Est, A. Der. Photoinduced Charge Separation in a Ferrocene-Aluminum(III) Porphyrin-Fullerene Supramolecular Triad. *Journal of Physical Chemistry B* **2010**, *114* (45), 14348–14357.

(10) Angiolillo, P. J.; Vanderkooi, J. M. Electron Paramagnetic Resonance of the Excited Triplet State of Metal-Free and Metal-Substituted Cytochrome c. *Biophys J* **1995**, *68* (6), 2505–2518.

(11) Kandrashkin, Y. E. ; D. V. M. ; van der E. A. Reversible Triplet Energy Hopping in Photo-Excited Molecules: A Two-Site Model for the Spin Polarization. *J. Chem. Phys.* **2020**, *153* (9), 094304.

(12) Salikhov, K. M. ; S. R. Z. ; B. A. L. *Spin Polarization and Magnetic Effects in Radical Reactions; Elsevier: Netherlands*; Elsevier: Netherlands, 1984.
